# Myeloid Cells Contribute to Tumor Lymphangiogenesis

**DOI:** 10.1371/journal.pone.0007067

**Published:** 2009-09-17

**Authors:** Adrian Zumsteg, Vanessa Baeriswyl, Natsuko Imaizumi, Reto Schwendener, Curzio Rüegg, Gerhard Christofori

**Affiliations:** 1 Institute of Biochemistry and Genetics, Department of Biomedicine, University of Basel, Basel, Switzerland; 2 Centre Pluridisciplinaire d'Oncologie, Lausanne Cancer Center, Epalinges, Switzerland; 3 Institute for Molecular Cancer Research, University of Zürich, Zürich, Switzerland; University of Calgary, Canada

## Abstract

The formation of new blood vessels (angiogenesis) and lymphatic vessels (lymphangiogenesis) promotes tumor outgrowth and metastasis. Previously, it has been demonstrated that bone marrow-derived cells (BMDC) can contribute to tumor angiogenesis. However, the role of BMDC in lymphangiogenesis has largely remained elusive. Here, we demonstrate by bone marrow transplantation/reconstitution and genetic lineage-tracing experiments that BMDC integrate into tumor-associated lymphatic vessels in the Rip1Tag2 mouse model of insulinoma and in the TRAMP-C1 prostate cancer transplantation model, and that the integrated BMDC originate from the myelomonocytic lineage. Conversely, pharmacological depletion of tumor-associated macrophages reduces lymphangiogenesis. No cell fusion events are detected by genetic tracing experiments. Rather, the phenotypical conversion of myeloid cells into lymphatic endothelial cells and their integration into lymphatic structures is recapitulated in two *in vitro* tube formation assays and is dependent on fibroblast growth factor-mediated signaling. Together, the results reveal that myeloid cells can contribute to tumor-associated lymphatic vessels, thus extending the findings on the previously reported role of hematopoietic cells in lymphatic vessel formation.

## Introduction

In the adult, the vascular network is usually expanded and remodeled by sprouting and proliferation of endothelial cells from pre-existing blood and lymphatic vessels, processes called angiogenesis and lymphangiogenesis, respectively. In addition to tissue resident cell types, several studies have demonstrated that BMDC are recruited to angiogenic sites to support the establishment of new vessels [1]–[3]. BMDC are typically sub-classified into hematopoietic progenitor cells (HPC) and endothelial progenitors cells (EPC). In various tumor models, HPC have been shown to contribute to blood vessel angiogenesis by secreting angiogenic factors and proteases required for the activation of latent forms of angiogenic factors [4], [5]. HPC have also been implicated in the preparation of a pre-metastatic niche in organs that are colonized by disseminating cancer cells [6]. EPC on the other hand have been shown to directly integrate into growing blood vessel walls, however, to varying extents, ranging from 0 to 50% and thus raising questions about their functional contribution to blood vessel angiogenesis in various physiological and pathological conditions [1], [7], [8]. Recently, it has been reported that also cells of the myeloid lineage are able to differentiate into *bona fide* blood endothelial cells [9].

Only few studies have addressed the role of BMDC in lymphangiogenesis. Hematopoietic stem cells (HSC) and BMDC have recently been shown to contribute to lymphatic endothelium in various organs and during embryonic development [10]–[12]. BMDC contribution to lymphatic vessels has also been reported under inflammatory conditions. For example, experiments employing a cornea angiogenesis model have revealed incorporation of BMDC in newly formed lymphatic vessels [13]. Furthermore, following rejection of human kidney transplants, lymphatic vessels within the rejected organs have been described to contain host-derived lymphatic endothelial cells, supporting the existence of bone marrow-derived lymphatic endothelial progenitor cells [14]. More specifically, myeloid cells present in the murine inflamed conjunctiva were found to express the lymphatic endothelial specific marker VEGFR-3 and to integrate into lymphatic structures that develop in mouse cornea transplants [15], [16]. In addition, macrophage depletion appeared to cause reduced lymphangiogenesis and impaired wound healing in diabetic mice [17].

The contribution of BMDC to tumor lymphangiogenesis is rather controversial. While two independent studies report a BMDC contribution to tumor lymphatics [10], [13], transplantation of Lewis Lung Carcinoma or B16-F1 melanoma cells in syngeneic mice has not revealed any integration of BMDC into newly formed lymphatic vessels [18]. Here, we have employed the Rip1Tag2 transgenic mouse model of pancreatic β cell carcinogenesis as well as subcutaneous transplantation of TRAMP-C1 murine prostate cancer cells in syngeneic C57Bl/6 mice to demonstrate that cells derived from the myeloid lineage can contribute to tumor lymphangiogenesis by integrating into tumor-associated lymphatic vessels. Moreover, *in vitro* culture assays reveal that macrophages can convert into lymphatic endothelial cells and integrate into cord-like structures formed by lymphatic endothelial cells. These data support and extend previous findings on the controversial role of hematopoietic cells in newly formed lymphatic vessels.

## Results

### BMDC integrate into tumor lymphatics

We have used the Rip1Tag2 (RT2) mouse model of multistage pancreatic β cell carcinogenesis to investigate the contribution of BMDC to tumor angiogenesis and lymphangiogenesis [19]. RT2 transgenic mice recapitulate hallmarks of tumor progression, including the regulated onset of tumor angiogenesis, the functional contribution of tumor-infiltrating immune cells to a pro-angiogenic tumor microenvironment, and the transition from adenoma to carcinoma [20]–[22]. When crossed to Rip1VEGF-C (VC) mice, double-transgenic RT2;VC mice develop tumors with high peritumoral lymphangiogenesis and lymph node metastasis [23].

**Figure 1:**
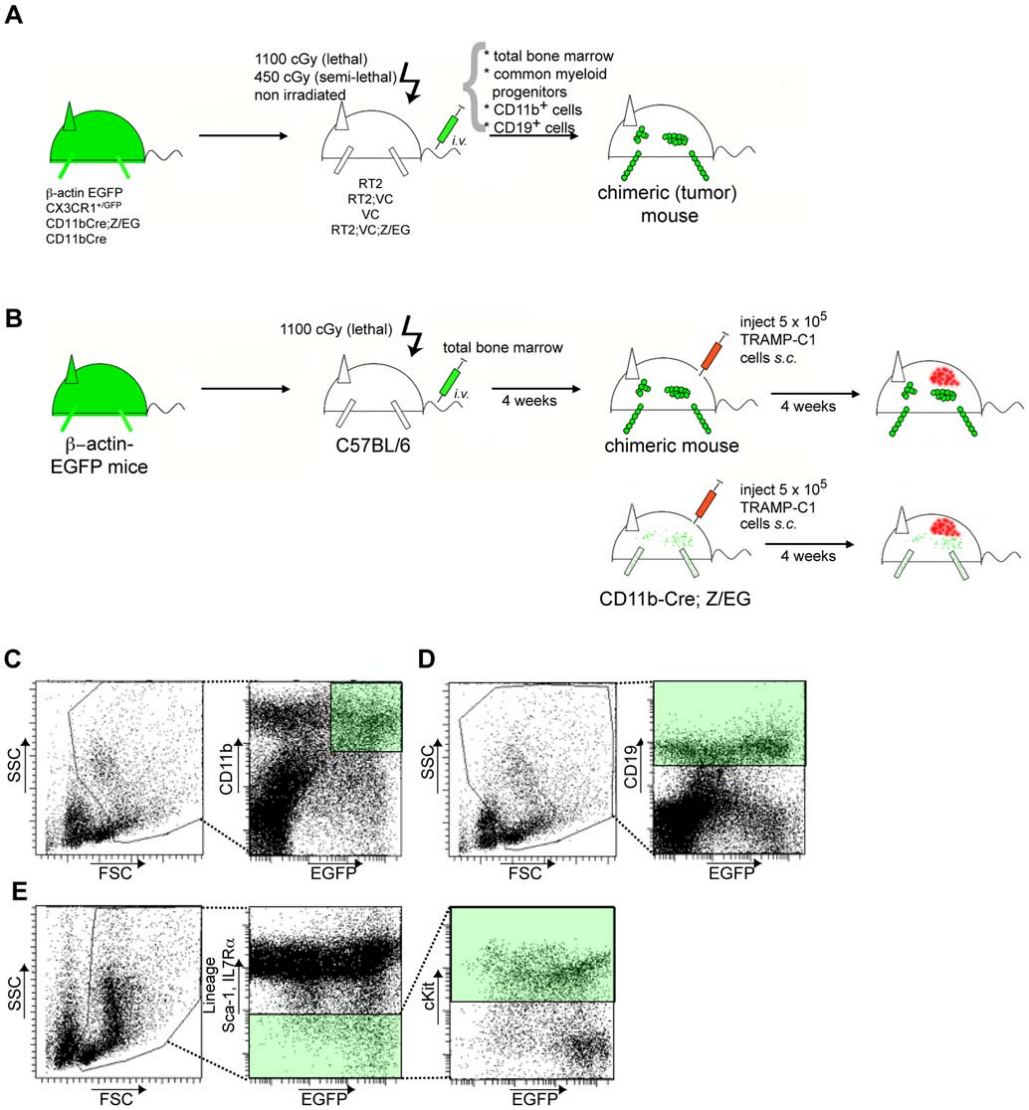
Bone marrow transplantation strategies. (A) For total bone marrow transplantations, 5×10^6^ T cell-depleted total bone marrow cells from donor mice were injected *i.v.* into lethally irradiated (2×550 cGy) mice, as indicated. Semi-lethally irradiated (450 cGy) mice were injected with FACS-sorted 4×10^5^ CD11b^+^ myeloid cells, 4×10^5^ CD19b^+^ B cells or 4×10^4^ common myeloid progenitors (CMP) cells. 4×10^5^ CD11b^+^ myeloid cells were also transferred into non-irradiated mice. After 3 weeks mice were sacrificed, engraftment of transplanted bone marrow was evaluated by FACS and pancreata were analyzed by histology for the presence of bone marrow-derived cells at the tumor site. (B) Schematic illustration of syngeneic TRAMP-C1 tumor experiments. 5×10^5^ TRAMP-C1 cells were injected into the flank of either C57BL/6 previously reconstituted with bone marrow of beta-actin-GFP transgenic mice or bone marrow of double-transgenic CD11b-Cre;Z/EG mice, and tumors were allowed to grow for 3 to 4 weeks. FACS analysis was used to assess bone marrow reconstitution or Cre recombinase-mediated GFP expression, respectively. Histological sections from TRAMP-C1 tumors were analyzed by immunofluorescence for the presence of GFP^+^ cells. (C–E) Flow cytometry-based strategy for cell sorting. (C) Within a scatter gate excluding lymphocytes, CD11b^high^/GFP^high^ cells were isolated by FACS. (D) CD19^+^ was used as marker for the isolation of B lymphocytes. (E) CMP cells were sorted as lin^−^/Sca-1^−^/IL7Rα^−^/cKit^+^ as described in Methods.

To investigate whether BMDC integrate into tumor blood and lymphatic vasculature in the RT2 model, lethally irradiated single transgenic RT2 and double-transgenic RT2;VC mice were transplanted with bone marrow isolated from actin-GFP transgenic mice (Figure 1A). FACS analysis of peripheral blood (PB) showed efficient hematopoietic reconstitution with more than 90% chimerism (data not shown). Immunofluorescence analysis of tumor sections revealed that the proportion of GFP^+^ tumor-infiltrating BMDC was invariant in the range of 3.5% of total cellularity, independent of the transplantation of single transgenic RT2 mice or double-transgenic mice expressing VEGF-C (Figure S1). From the GFP^+^ BMDC within the tumors, approximately 80% were F4/80^+^ macrophages (Figure S1). Immunofluorescence co-staining for F4/80 and the hyualuronan receptor LYVE-1 identified LYVE-1^+^ macrophages in the tumor periphery with relatively large size compared to intra-tumoral macrophages (data not shown) [24], [25]. In contrast, Podoplanin or Prox-1 were not expressed by these tumor-associated macrophages (TAM). These observations instructed us to carefully differentiate between tumor lymphatic endothelium, defined as a continuous LYVE-1^+^ vessel lining, and isolated, peritumoral LYVE-1^+^ TAM.

The potential contribution of BMDC to intra-tumoral blood vessels was analyzed by confocal microscopy and subsequent 3D reconstitution on pancreatic sections of transplanted RT2 and RT2;VC mice stained for the endothelial marker CD31 and for GFP. Bone marrow-derived, GFP^+^ cells were mainly found in close proximity of tumor blood vessels, yet a significant direct incorporation of BMDC into the blood vasculature was not detectable (data not shown).

**Figure 2:**
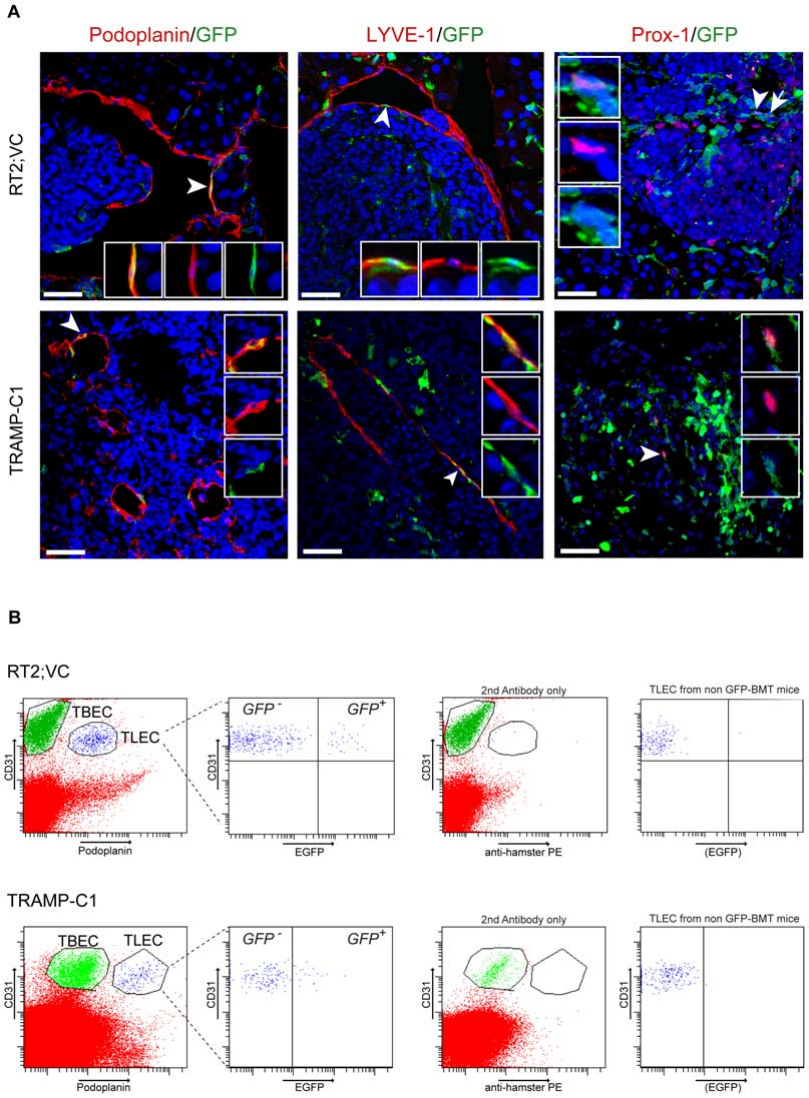
BMDC integrate into tumor-associated lymphatic vessels. (A) Lethally irradiated RT2;VC mice (5 mice) were reconstituted with GFP-labeled bone marrow. 20 µm histological pancreatic sections were stained for the lymphatic markers Podoplanin, Prox-1, LYVE-1 and for GFP as indicated and analyzed by confocal microscopy and subsequent 3D reconstitution. Representative tumor sections per lymphatic marker are shown. 3% of Podoplanin^+^ TLEC (7 Podoplanin^+^/GFP^+^ cells out of 227 Podoplanin^+^ cells) as well as 3.5% of Prox-1^+^ or LYVE-1^+^ TLEC (14 Prox-1^+^/GFP^+^ cells out of 400 Prox-1^+^ cells and 17 LYVE-1^+^/GFP^+^ cells out of 485 LYVE-1^+^ cells) are bone marrow-derived. TRAMP-C1 tumors were subcutaneously implanted in C57BL/6 mice (4 mice) previously reconstituted with GFP-labeled bone marrow. 7–20 µm histological tumor sections were stained as described above. 4.1% of Podoplanin^+^ TLEC (14 Podoplanin^+^/GFP^+^ cells out of 334 Podoplanin^+^ cells) as well as about 2.8% of LYVE-1^+^ TLEC (11 LYVE-1^+^/GFP^+^ cells out of 395 LYVE-1^+^ cells) are bone marrow-derived. Arrows indicate double-positive cells and arrowheads indicate double-positive cells shown in inset magnifications. Insets show merged and individual channels. DAPI stains nuclei (blue). Scale bars: 40 µm. (B) Tumors of GFP-labeled bone marrow-transplanted RT2;VC mice or TRAMP-C1 tumors grown in GFP-labeled bone marrow-transplanted C57BL/6 mice were enzymatically digested (3 mice each). Single cell suspensions were stained for the pan-endothelial marker CD31 and the lymphatic endothelial marker Podoplanin and analyzed by FACS (left panels). 9.4+/−4.1% (RT2;VC) and 10+/−4.6% (TRAMP-C1) of CD31^+^/Podoplanin^+^ TLEC were GFP^+^, indicating their bone marrow origin (middle left panels). As control, the anti-Podoplanin antibody was omitted resulting in no separation between TLEC and TBEC (middle right panels). Furthermore, similar analysis of tumors grown in non-transplanted mice showed no GFP^+^ cells within TLECs (right panels).

**Figure 3:**
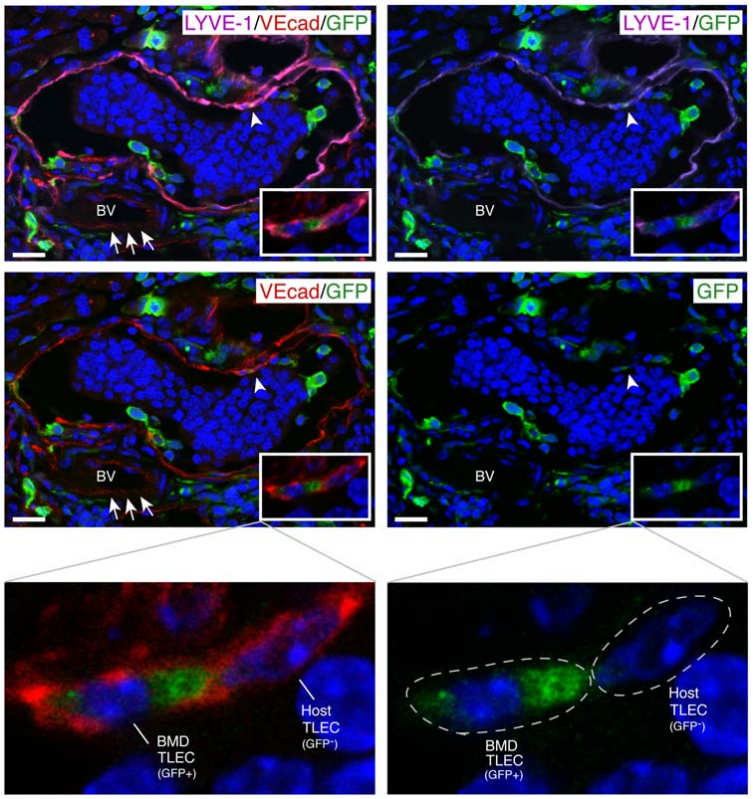
BMDC integrated into tumor lymphatics express vascular endothelial cadherin. A representative tumor section from RT2;VC mice previously reconstituted with GFP-labeled bone marrow was stained for the lymphatic marker LYVE-1 (purple), for the cell junction molecule VE-cadherin (red), and for GFP (green) and analyzed by confocal microscopy and subsequent 3D reconstitution. As indicated by arrowheads and shown magnified in insets, VE-cadherin expression, indicative for homophilic cell-cell contact, is observed between bone marrow-derived (BMDTLEC) and host-derived TLEC (HTLEC). Note the continuous VE-cadherin staining between lymphatic endothelial cells in contrast to the cell-cell contact restricted staining of blood endothelial cells depicted by arrows. DAPI was used for nuclear counterstaining (blue). The DAPI-staining cells within the lymphatic vessel represent a cluster of disseminated tumor cells. BV: blood vessel. Scale bars: 20 µm.

In contrast, BMDC had incorporated into lymphatic vessels surrounding VEGF-C expressing β cell tumors of transplanted RT2;VC mice. Pancreatic sections from these mice were stained for the three lymphatic markers Podoplanin, Prox-1 and LYVE-1 and for GFP. Confocal imaging revealed that 3% of Podoplanin^+^ tumor lymphatic endothelial cells (TLEC) as well as 3.5% of Prox-1^+^ or LYVE-1^+^ TLEC co-expressed GFP, indicating that approximately 3% of tumor-surrounding lymphatic endothelial cells are derived from the bone marrow (Figure 2A). Routine 3D reconstitution analysis by compiling the Z-stacks of the confocal images enabled us to distinguish integrated GFP^+^ BMDC cells from cells located in the close vicinity of lymphatic vessels or transmigrating through the lymphatic endothelial barrier, as shown in Supplemental Movie S1 and S2. Furthermore, VE-cadherin, an endothelial-specific adherens junction molecule reported to connect lymphatic endothelial cells in lymphatic vessels [26], was expressed on host as well as on bone marrow-derived TLEC, further demonstrating a functional integration of BMDC into tumor lymphatic vasculature (Figure 3). Note that in contrast to blood endothelial cells, where VE-cadherin principally clusters at cell-cell junctions (Figure 3, arrows), VE-cadherin staining on lymphatic endothelium was more homogenously distributed throughout the membrane [26].

To assess the general significance of the findings in the RT2 insulinoma model as well as to test whether the observed integration of BMDC occurred also in the absence of transgenic expression of VEGF-C, we employed the TRAMP-C1 murine prostate adenocarcinoma cell line previously shown to induce robust tumor lymphangiogenesis upon transplantation into syngeneic C57Bl/6 mice [27], [28]. TRAMP-C1 cells were injected *s.c.* into one flank of C57Bl/6 mice that had been previously transplanted with GFP^+^-labeled bone marrow (Figure 1B). In the resulting tumors, the number and morphology of BMDC that had integrated into tumor lymphatic vessels were comparable to the results obtained with RT2;VC mice. GFP^+^ cells were detected in lymphatic vessels staining for LYVE-1 and Podoplanin (Figure 2A) and constituted 2.8% of LYVE-1^+^ and 4.1% of Podoplanin^+^ cells within lymphatic vessel structures. GFP expression was also detected in Prox-1^+^ TLEC (Figure 2A), however to a lower extent as compared to LYVE-1 or Podoplanin. This might be explained by the fact that overall only a subset of LYVE-1^+^ TLEC express Prox-1 (data not shown).

To corroborate the simultaneous expression of lymphatic markers and GFP in individual cells, single cell suspensions from tumors of GFP^+^ bone marrow-transplanted or control non-transplanted mice were analysed by FACS (Figure 2B). TLEC were identified by co-expression of CD31 and Podoplanin (Figure 2B, left panels; note that similar to blood vessel endothelial cells TLEC express CD31, albeit at slightly reduced levels). In tumors derived from RT2;VC and TRAMP-C1 mice, 9.4+/−4.1% and 10+/−4.6% of TLEC, respectively, were GFP^+^, confirming the immunofluorescence data. As expected, GFP^+^ TLEC could not be observed in non-transplanted mice (Figure 2B, right panels). In order to avoid detecting false positives by cell duplets containing GFP^+^ BMDC and TLEC that would appear as CD31^+^/Podoplanin^+^/GFP^+^ triple-positive, such events were rigidly excluded by forward scatter pulse width (data not shown).

We next investigated whether BMDC integration into newly formed lymphatic structures occurred only in a tumor microenvironment by transplanting non tumor-bearing, single-transgenic VC mice with GFP-labeled bone marrow. Notably, no GFP^+^ cells were found incorporated into the lymphatic vessels surrounding normal islets of Langerhans in these mice [23] (Figure S2). These results demonstrate that in the experimental systems investigated here, BMDC only incorporate into tumor-associated- lymphatic vessels but not into newly forming lymphatic vessels of normal tissue.

### Integrated BMDC are of myeloid origin

**Figure 4:**
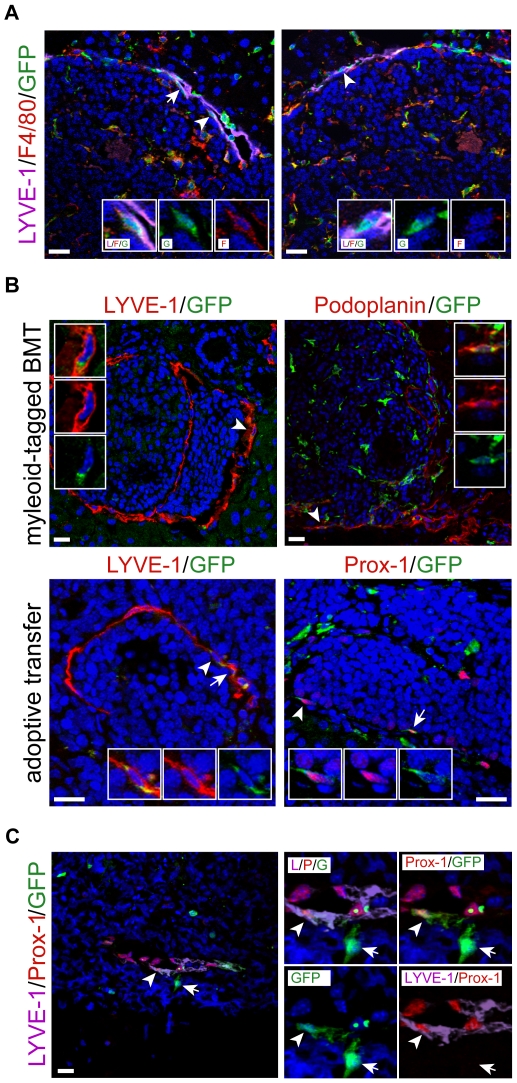
Myeloid origin of bone marrow-derived TLEC. (A) 20 µm histological pancreatic sections of GFP-labeled bone marrow-transplanted RT2;VC mice were stained for the lymphatic marker LYVE-1 (purple), for the macrophage marker F4/80 (red), and for GFP (green) as indicated and analyzed by confocal microscopy and subsequent 3D reconstitution. Two representative tumor sections are shown. Left panel: BMDC that have integrated into tumor lymphatics express the macrophage marker F4/80. Arrows indicate triple positive cells and the arrowhead indicate a triple-positive cell shown in inset magnifications. Right panel: not all integrated cells express F4/80. The arrowhead indicates an integrated cell (LYVE-1^+^/GFP^+^) that does not express F4/80. Inset magnifications of this double-positive cell are shown. Insets show merged and individual channels. (B) 20 µm histological pancreatic sections of RT2;VC mice transplanted with bone marrow isolated from either CX3CR1^+/GFP^ (2 mice) or CD11bCre;Z/EG mice (3 mice), or adoptively transferred with FACS-sorted CD11b^+^ cells were stained for the lymphatic markers LYVE-1, Podoplanin or Prox-1 (red) as well as for GFP (green) and analyzed by confocal microscopy. Representative tumor sections are shown. In both transplantation settings, myeloid cells were found integrated into the lymphatic vasculature surrounding the tumors. Arrowheads indicate double-positive cells shown in inset magnifications (first inset: merged channels, second and third insets: red and green channel, respectively). DAPI stains nuclei (blue). Scale bars: 20 µm. (C) CD11b^+^ lineage tracing experiments demonstrate the myeloid origin of TLEC in a transplantation-independent setting. TRAMP-C1 tumors were grown subcutaneously in CD11b-Cre;Z/EG mice (3 mice). In these mice, cells that have passed through a CD11b^+^ myeloid lineage express GFP. Fluorescent triple staining of histological tumor sections reveal co-expression of LYVE-1 and Prox-1 by myeloid-derived GFP^+^ cells integrated into lymphatic vessels (arrowhead). The right panel represents a magnification of the relevant region with individual channels combined. Note that GFP^+^ cells not connected to vascular structures do express neither LYVE-1 nor Prox-1 (arrow). DAPI stains nuclei (blue). Scale bars: 20 µm.

Myeloid cells have been reported to give rise to blood endothelium and, under inflammatory conditions, to lymphatic endothelium [9], [16], [17]. To investigate whether BMDC contributing to tumor lymphangiogenesis express macrophage markers, pancreatic sections of transplanted RT2;VC mice were stained by immunofluorescence for the lymphatic marker LYVE-1, the macrophage marker F4/80 and GFP (Figure 4A). Triple-positive GFP^+^/LYVE-1^+^/F4/80^+^ cells were readily observed within the lymphatic vessel lining surrounding the tumors. Interestingly, not all BMDC that had integrated into the lymphatic vasculature expressed F4/80, suggesting that macrophages physically contributed to tumor lymphatics but eventually lost their macrophage features upon integration, as previously reported [17].

Next, we performed various independent lineage-tracing experiments to assess whether cells of the myeloid lineage were indeed able to incorporate into tumor lymphatic vessels. First, lethally irradiated RT2;VC mice were transplanted with bone marrow isolated from either CX3CR1^+/GFP^ mice or CD11b-Cre;Z/EG mice (Figure 1A). In CX3CR1^+/GFP^ mice, the coding region for EGFP had been inserted in the *CX3CR1* gene, a receptor expressed mainly by monocytes and to a minor extent by a subset of lymphocytes, resulting in monocyte-specific GFP expression [29] (Figure S3A). The Z/EG transgene contains, under the control of an ubiquitous promoter, a *lacZ* gene/stop cassette flanked by loxP recombination sites and followed by EGFP [30]. When crossed to CD11b-Cre mice, expressing Cre recombinase under the control of the myeloid specific CD11b promoter, Cre-mediated excision of the *lacZ* gene/stop cassette induced permanent GFP expression exclusively in cells having passed through a CD11b-positive, myeloid stage [31]. Pancreatic sections of transplanted RT2;VC were stained for the lymphatic markers Podoplanin or LYVE-1 and for GFP, and double-positive cells were scored. In both transplantation settings, GFP^+^ cells were found integrated into the tumor lymphatic vasculature, demonstrating that cells of the myeloid lineage physically contributed to tumor lymphangiogenesis (Figure 4B, upper panels).

We also tested whether CD11b^+^ cells integrated into tumor lymphatics without prior bone marrow transplantation by transplanting TRAMP-C1 cells into CD11b-Cre;Z/EG mice (Figure 1B). Specific Cre-mediated recombination within the myeloid lineage of these mice was confirmed by FACS analysis of PB cells (Figure S3B). In the resulting tumors, GFP^+^ cells were found incorporated into LYVE-1^+^ and Podoplanin^+^ lymphatic vessel lining (data not shown). Triple-staining for LYVE-1, Prox-1 and GFP further showed that formerly myeloid cells expressed two lymphatic markers simultaneously (Figure 4C, arrowhead), indicating a significant differentiation towards a lymphatic endothelial phenotype. These results also revealed that integration occurred independently of prior irradiation, which had been previously reported to increase macrophage infiltration in human cancer [32].

In a second series of lineage-tracing experiments, FACS-sorted CD11b^+^/GFP^+^ cells were *i.v.* injected into semi-lethally or non-irradiated RT2;VC mice (Figure 1A and C). 3 weeks after injection, adoptively transferred GFP^+^ cells were observed integrated into tumor lymphatics, identified by LYVE-1 or Prox-1 expression (Figure 4B, lower panels). The fact that the adoptive transfer of CD11b^+^/GFP^+^ cells into non-irradiated RT2;VC mice resulted into an integration of the injected cells into tumor lymphatics indicates that a full reconstitution of the hematopoietic system by stem cells is not a prerequisite for BMDC contribution to tumor lymphangiogenesis.

To assess whether common myeloid progenitor cells (CMP) [33] provide the cells that incorporate into tumor lymphatics, FACS-sorted CMP cells (lin^−^/Sca-1^−^/IL7Rα^−^/cKit^+^/GFP^+^; Figure 1E) were adoptively transferred into semi-lethally irradiated RT2;VC mice. FACS analysis of PB cells 3 weeks post-injection revealed that transplanted CMP contributed to the generation of CD11b^+^/F4/80^+^ monocytes and CD11b^+^/F4/80^−^ granulocytes but not to CD19^+^ B lymphocytes or CD3^+^ T lymphocytes (Figure S3C). Also here, GFP^+^ cells were found integrated into tumor-associated lymphatic endothelium, detected by LYVE-1 or Podoplanin expression (Figure S4). In contrast, adoptive transfer of FACS-sorted CD19^+^/GFP^+^ B cells (Figure 1A and D) did not result in any incorporation of these cells into tumor lymphatic vessels (Figure S5), underscoring the exclusive ability of myeloid cells to contribute to tumor lymphangiogenesis and excluding the possibility that minor contaminations of hematopoietic stem cells in the FACS-sorted fractions may have contributed to the GFP^+^ cells that incorporated into tumor lymphatics. Finally, FACS analysis of tumors from non-transplanted RT2;VC mice revealed that some *bona fide* CD31^+^/LYVE-1^+^ TLEC express the myeloid marker CD11b (Figure S6), indicating that the integration of cells of the myeloid lineage into tumor lymphatics and their simultaneous expression of lymphatic endothelial cell markers occurs also in the absence of any bone marrow transplantation.

In order to assess potential fusion events between bone marrow-derived cells and pre-existing lymphatic endothelial cells, lethally irradiated triple-transgenic RT2;VC;Z/EG mice were transplanted with bone marrow isolated from CD11b-Cre mice (Figure 1A). Fusion of CD11b^+^-BMDC, expressing the Cre recombinase, with host (tumor lymphatic endothelial) cells would result in GFP expression from the recombined Z/EG locus. Seven weeks after transplantation, no GFP^+^ cells were detected in or around lymphangiogenic insulinomas, indicating that Cre-expressing, bone marrow-derived myeloid cells had not fused with RT2;VC;Z/EG lymphatic endothelial cells or any other host cell (data not shown).

These results demonstrate that cells found integrated into growing tumor lymphatic vessels can have a myeloid origin and that bone marrow-derived lymphatic progenitor cells are at least in part derived from the already myeloid committed hematopoietic lineage.

### Depletion of macrophages

**Figure 5:**
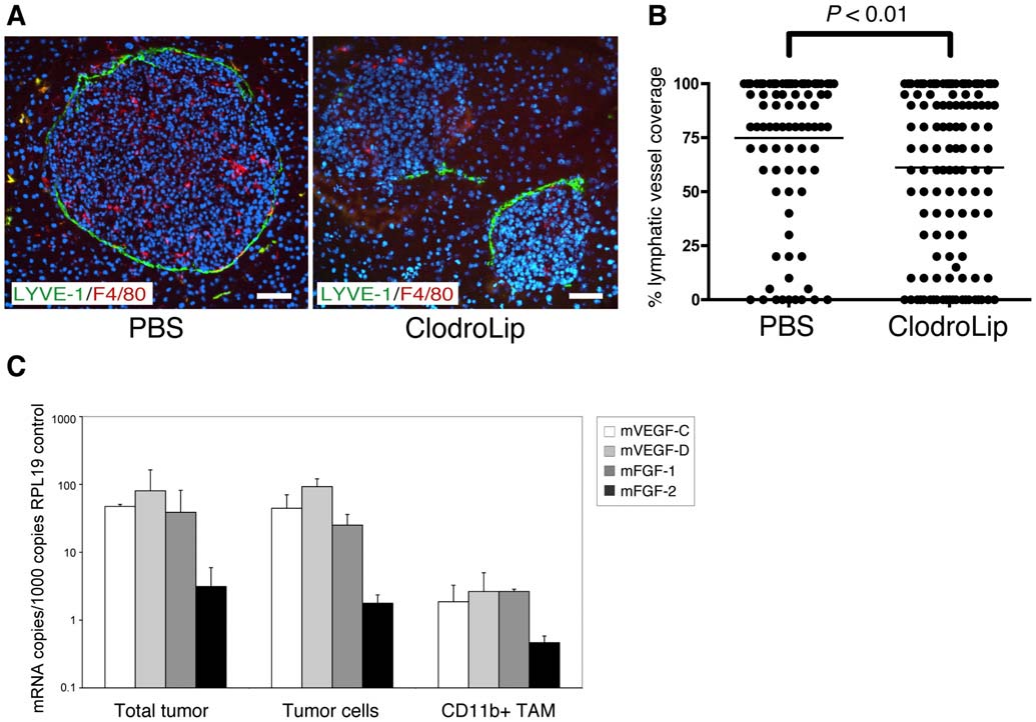
Depletion of macrophages reduces peritumoral lymphatic vessel density. (A) RT2;VC mice were treated with liposome-encapsulated Clodronate (ClodroLip). Pancreatic sections representing a total of 5 PBS vehicle control-treated mice (97 tumors) and 6 ClodroLip-treated (132 tumors) mice were analyzed. Successful depletion of intra- and extra-tumoral macrophages in ClodroLip-treated mice is illustrated by the reduction of F4/80 immunoreactivity (red). Co-staining with the lymphatic endothelial marker LYVE-1 (green) reveals a reduced coverage of tumors by lymphatic vessels in ClodroLip-treated mice vs. in PBS-treated mice. DAPI was used for nuclear counterstaining (blue). T: tumor. Scale bar: 50 µm. (B) Tumors of ClodroLip and control-treated mice were analyzed by immunofluorescence staining with antibodies against LYVE-1 for the extent of lymphatic vasculature surrounding the perimeter of the tumors. Tumors of control-treated mice were surrounded by 90% or more with lymphatic vessels (median 90%, mean 74.9%), whereas tumors of ClodroLip-treated mice had significantly lower coverage (median 70%, mean 61.1%; P<0.01, Mann-Whitney test). (C) Tumor-associated CD11b^+^ macrophages (TAM) and tumor cells were isolated from tumors of RT2;VC mice by flow cytometry, and mRNA levels for murine VEGF-C, VEGF-D, FGF-1 and FGF-2 were determined using quantitative RT-PCR and compared to levels in total tumors. Shown is the result of three independent cell isolations. ΔCT values have been calculated and normalized to internal control (RPL19) CT value. The results are displayed as mRNA copies per 1000 copies of control RPL19 mRNA to visualize the relative levels of mRNA of interest to internal RPL19 control mRNA.

To investigate the functional contribution of macrophages to tumor lymphangiogenesis, RT2;VC mice were treated with liposome-encapsulated Clodronate (ClodroLip) or PBS as vehicle-control for 4 weeks to ablate TAM [34], [35]. Successful macrophage depletion was achieved as shown by reduced F4/80 immuno-reactivity in ClodroLip treated mice (Figure 5A). Peri-tumoral lymphatic vessel density (LVD) was significantly decreased in ClodroLip vs. PBS treated mice (Figure 5B; treated: median 70%, mean: 61% vs. control: median 90%, mean 74.9%; P<0.01). Notably, the formation of lymph node metastasis was not affected by the significant but rather moderate reduction of tumor lymphangiogenesis (data not shown). In contrast to a recent study where ClodroLip reduced tumor growth of xenotransplants in immuno-compromised mice [36], average tumor volume, tumor incidence and blood vessel density were not significantly reduced in our experiments (Figure S7). To evaluate the amount of VEGF-C, VEGF-D, FGF-1 and FGF-2 provided by TAM, CD11b^+^ cells were FACS-isolated from RT2;VC tumors and mRNA levels were assessed by quantitative RT-PCR and compared to levels in total tumors and FACS-isolated tumor cells. The expression of endogenous murine VEGF-C, VEGF-D, and FGF-1 in total tumors and tumor cells (not considering the high levels of transgenic human VEGF-C expression in RT2;VC mice) was higher than in TAM (Figure 5C). FGF-2 was not found expressed at significant levels in any of the samples, surprisingly not even in FACS-sorted CD11b+ TAM. However, the lack of FGF-2 expression is not unexpected, since FGF-2 expression has been repeatedly found to be very low to undetectable in Rip1Tag2 tumors (and thus also in infiltrating macrophages). From these results we conclude that macrophages contribute to tumor lymphangiogenesis in RT2;VC mice by processes other than the secretion of main lymphangiogenic factors.

### Macrophages form and contribute to lymphatic-like structures in vitro

**Figure 6:**
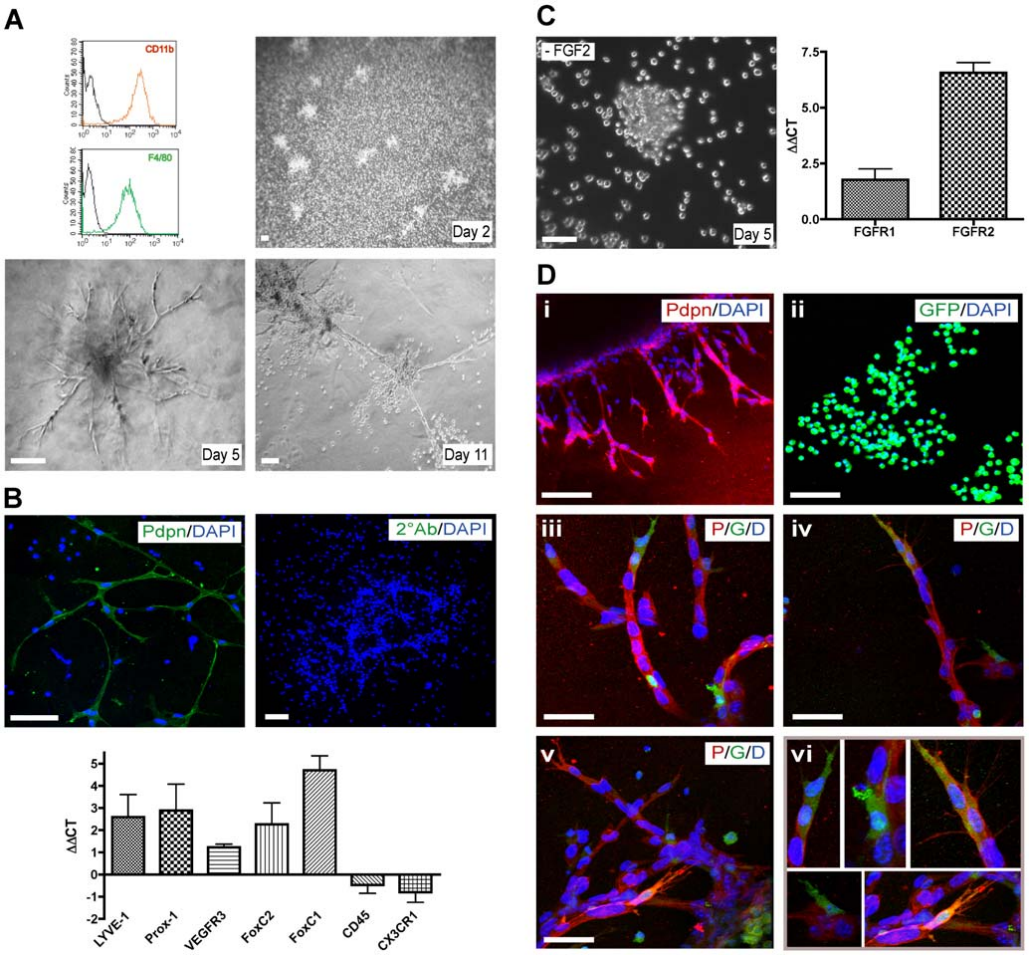
Bone marrow-derived-macrophages form and contribute to lymphatic-like structures *in vitro.* (A) *In vitro* generated macrophages showed a specific marker expression profile (CD11b^+^/F4/80^+^) (upper left panel). Tube formation on Matrigel was monitored by phase-contrast microscopy. At day 2, macrophages formed clusters. Between days 3 and 15, they developed into cord-like structures with numbers of branches increasing over time. Scale bar: 100 µm. (B) Immunofluorescence staining against Podoplanin (Pdpn) revealed that macrophages having formed cord-like structures express Podoplanin whereas single cells do not. Staining of the tubular structures in the absence of any primary antibody was used as a control (2°Ab). DAPI stains nuclei (blue). Scale bar: 100 µm. Quantitative RT-PCR analysis revealed that upon tube formation, macrophages up-regulate lymphatic markers (LYVE-1, Prox-1, VEGFR-3, Foxc2, Foxc1) and down-regulate hematopoietic/myeloid marker (CD45, CX3CR1). ΔΔCT corresponds to the difference between the normalized CT values of macrophages forming tubes (day 8) and macrophages not having yet formed tubes (day 1). (C) FGF-2 is required for the formation of cord-like structures by macrophages, as its specific exclusion from culture medium abrogated this process (left panel). Furthermore, analysis of mRNA levels revealed up-regulation of FGF receptors -1 and 2 during cord formation. (D) Immortalized Podoplanin^+^ murine lymphatic endothelial cells (SV-LEC) (i), GFP-labeled bone marrow-derived-macrophages (ii), and mixed cultures of macrophages and SV-LEC (iii-vi) were seeded in Matrigel. At day 5, cells were stained for Podoplanin (red) and analyzed by confocal microscopy. Mixed cultures demonstrate that bone marrow-derived macrophages contribute to SV-LEC-mediated cord formation: GFP^+^ cells (green) are found integrated into Podoplanin^+^ cord-like structures (iii–vi). Note the preferential integration of bone marrow-derived macrophages at the tips and branch points of sprouting cord-like structures formed by SV-LEC (magnified in panel vi). DAPI stains nuclei (blue). Scale bars: 100 µm (i–ii) and 50 µm (iii–vi).

We next investigated whether bone marrow-derived-macrophages had an intrinsic capability to form lymphatic vessel-like structures. Bone marrow cells were cultured for 7 days in 30% M-CSF containing-medium to induce the specific differentiation of progenitor cells into non-activated macrophages [37]. Flow cytometric analysis confirmed the macrophage identity (CD11b^+^/F4/80^+^) of these cells (Figure 6A). The bone marrow-derived-macrophages were then activated with LPS and seeded on Matrigel to monitor differentiation and tube formation. After two days in endothelium-specific medium supplemented with defined growth factors, macrophages associated in clumps, before forming cord-like structures with increasing connections between days 3 and 15 (Figure 6A). Confocal immunofluorescence microscopy analysis at day 12 revealed that only macrophages that had formed cord-like structures and not single isolated cells expressed the lymphatic marker Podoplanin (Figure 6B). Furthermore, quantitative RT-PCR analysis of mRNA from macrophages isolated either before or after the cord formation process revealed a marked up-regulation of the lymphatic markers LYVE-1, Prox-1, VEGFR-3, FoxC2 and FoxC1 as well as a down-regulation of the hematopoietic/monocytic markers CD45 and CX3CR1 during cord formation (Figure 6B). Exclusion of individual growth factors revealed the requirement of FGF-2 for cord formation (Figure 6C), whereas the other supplemental growth factors (VEGF-A, IGF-1, EGF, hydrocortisone) were dispensable. Accordingly, mRNA levels of FGF receptor-1 and 2 were up-regulated during cord formation, as revealed by quantitative RT-PCR analysis (Figure 6C).

In order to explore the capacity of myeloid cells to integrate into lymphatic structures *in vitro*, GFP-labeled macrophages were generated as described above from bone marrow of actin-GFP transgenic mice and subsequently cultured on Matrigel alone or in combination with SV40 T antigen-immortalized murine lymphatic endothelial cells (SV-LEC) [38]. Five days later, the cultures were stained for Podoplanin. Cultured SV-LEC formed cord-like structures positive for Podoplanin expression (Figure 6D/i), and cultured *in vitro* activated macrophages were positive for GFP (Figure 6D/ii). In mixed cultures, bone marrow-derived macrophages lined up with SV-LEC, incorporating into cord-like structures and expressed Podoplanin (Figure 6D/iii–vi). Interestingly, GFP^+^ macrophages were predominantly located at the tips and at branch points of growing cord-like structures (Figure 6D/iii–vi) and seemed to guide SV-LEC to form a new sprout as observed by time-lapse video microscopy (Figure S8, Supplemental Movie S3). The live visualization of GFP+ macrophages guiding LEC together with the observation that macrophages located at the tip of the lymphatic sprout exhibit filopodia-like structures (Figure 6D/iii–vi) strongly suggest that instead of capping the exposed ends, they actively instigate the new sprout.

These results demonstrate that bone marrow-derived macrophages have the ability to form lymphatic tube-like structures *in vitro*, a process requiring FGF signaling. Their preferred incorporation at tips and branchpoints of pre-existing lymphatic cord-like structures data suggest a role of macrophages in lymphatic endothelial cell sprouting.

## Discussion

Research on BMDC in patho-physiological processes, such as atherosclerosis, limb/heart ischemia and cancer, has in the past mainly focused on the importance of hematopoietic cells in promoting or attenuating inflammation, in clearing cancer cells, or in inducing immunological tolerance to neoplastic lesions. However, recent findings indicate that the bone marrow is also a rich source of progenitor cells with mesenchymal and endothelial potential [39], [40]. In the case of endothelial progenitor cells, the lineage relationship to the hematopoietic system is not clear. While some experiments have recently revealed that during development hematopoietic cells arise from a specialized endothelium named the hemogenic endothelium [41]–[43], other reports provide evidence that the reverse direction of cellular conversion is also possible, i.e. that myeloid cells can contribute to the formation of blood endothelial cells [9], [44].

Here, we have used bone marrow transplantation experiments in two different mouse models of carcinogenesis to demonstrate that BMDC significantly contribute to tumor lymphangiogenesis, but rarely integrate into tumor blood vessels. We have performed lineage-tracing experiments to obtain insights into the ontogeny of bone marrow-derived TLEC. First, transplantations of FACS-sorted bone marrow fractions representing different hematopoietic lineages or of total bone marrow expressing GFP under a myeloid specific promoter indicate that integrated BMDC are derived from the myeloid lineage. Second, genetic tagging of myeloid cells with GFP confirms this notion; cells that have passed through the myeloid lineage are found integrated into the lymphatic vasculature surrounding tumors. Third, depletion of tissue macrophages using ClodroLip significantly reduces peri-tumoral lymphatic vessel density, demonstrating a functional role of macrophages in tumor lymphangiogenesis. Fourth, the intrinsic ability of myeloid cells to give rise and incorporate into lymphatic-like structures is recapitulated in two *in vitro* assays. Taken together, *in vitro* and *in vivo* experimentation strongly suggest that cells of the myeloid lineage physically contribute to tumor lymphangiogenesis.

The statement that BMDC can also contribute to lymphangiogenesis in a paracrine-independent manner is highly debated. As with any controversial scientific discussion, well-controlled studies conducted in different laboratories and leading to similar conclusions constitute the basis to overcome skepticism. Along these lines, the present study is consistent with previously described observations that hematopoietic cells can contribute to lymphatic endothelium, in normal organs, during embryonic development, in inflammatory conditions, and in a tumor microenvironment [10]–[17]. The experimental results presented here extend these findings by identifying that cells of the myeloid lineage can contribute to lymphatic endothelium in a tumorigenic context.

The existence of specific lymphatic progenitor cells (LPC), distinct from hematopoietic as well as blood endothelial progenitor cells, has not been established. Based on a number of control experiments, such as the transplantation of FACS-sorted CD19^+^ B cells or the adoptive transfer of CD11b^+^ myeloid cells into non-irradiated recipients, we exclude the possibility that FACS-sorted cell fractions may have contained hematopoietic stem cells that also reconstitute potential LPC. Rather, our data indicate a myeloid origin of cells that integrate into tumor-associated lymphatic endothelial cells, thus supporting the notion that LPC reside at least partially within an already committed hematopoietic lineage. It is interesting to note that the myeloid contribution to lymphatic vessels has thus far only been described to occur under inflammatory conditions, such as corneal transplantation and wound healing [16], [17]. In contrast, the existence of LPC within the HSC population, but distinct from the myeloid lineage, has been reported to play a role in steady state lymphangiogenesis in normal liver, stomach, and intestine of HSC-transplanted mice [10]. The contribution of hematopoietic cells to lymphangiogenesis has been also shown during embryonic development. Mice lacking the hematopoietic signaling molecules SLP-76, Syk and PLCγ2 fail to separate emerging lymphatic vessels from blood vessels [11], [45]. Notably, this phenotype depends on the expression of these signaling molecules in hematopoietic progenitor cells that give rise to circulating endothelial progenitor cells, thus demonstrating a cell-autonomous contribution of hematopoietic cells to vascular development [11], [12].

The low frequency of bone marrow-derived lymphatic endothelial cells is a recurrent observation among the different studies, raising questions towards the functional contribution of these cells to lymphangiogenesis. However, the pharmacological depletion of tumor-associated macrophages results in a decrease in lymphangiogenesis [16] (Figure 5). Moreover, our results indicate that macrophages are not the main source of lymphangiogenic factors in the RT2 tumor model, leading us to conclude that macrophages contribute to tumor lymphangiogenesis, at least in this model, by processes other than the paracrine secretion of lymphangiogenic factors. Rather, when co-cultured *in vitro* with lymphatic endothelial cells, bone marrow-derived macrophages incorporate predominantly at the tips and branch points of growing cord-like structures. *In vitro* time-lapse video microscopy confirms this notion and shows that macrophages, after being recruited to lymphatic endothelial cells, are able to instigate lymphatic sprouts. These observations suggest that myeloid-derived lymphatic endothelial cells may exert a specific functional role, which may explain the need of only a low number of these cells for the complete process of lymphangiogenesis.

In summary, we demonstrate here that in the context of tumor growth, cells of the myeloid lineage can contribute to the formation of tumor-associated lymphatic endothelium. Since tumor lymphatic vessels provide a route for metastatic dissemination, understanding the functional role of bone marrow-derived tumor lymphatic endothelial cells seems warranted.

## Methods

### Mouse strains

Generation and phenotypic characterization of Rip1Tag2, Rip1VEGF-A and Rip1VEGF-C mice have been described previously [19], [23], [46]. C57BL/6-Tg(ACTB-EGFP)mice [47] and Z/EG mice [30] were provided by K. Hafen (University of Basel). CD11b-Cre mice [31] and CX3CR1^+/GFP^ mice [29] were obtained from J. Vacher (University of Montreal) and C. Rüegg (CePO Lausanne), respectively. All experiments involving mice were performed in accordance with the guidelines of the Swiss Federal Veterinary Office (SFVO) and the regulations of the Cantonal Veterinary Office of Basel-Stadt.

### Total bone marrow transplantations

Bone marrow cells were extracted under sterile conditions from femurs and tibiae from donor mice indicated in Figure 1. After T cell depletion [48], 5×10^6^ cells were injected in the tail vein of lethally irradiated (2×550 cGy) 6 week old mice which were sacrificed for further analysis 5 to 7 weeks after transplantation.

### TRAMP-C1 subcutanous tumor model

5×10^5^ TRAMP-C1 cells [49] (provided by N. Greenberg, FHCRC, Seattle) were injected into the flank of either GFP-labeled bone marrow transplanted C57BL/6 mice (4 weeks after transplantation) or CD11b-Cre;Z/EG mice and grown for 3 to 4 weeks.

### Flow cytometric analysis

Cells were washed in PBS supplemented with 5% FBS, Fc-blocked with a monoclonal antibody against mouse CD16/CD32 (Clone 2.4G2, Pharmingen), and stained with directly-labeled monoclonal antibodies against mouse CD19 (Clone MB19-1, eBioscience), CD3 (Clone 145-2C11, eBioscience), CD11b (Clone M1/70.15, CALTAG), F4/80 (Clone CI:A3-1, Serotec), LYVE-1 (Clone ALY7, CliniSciences), CD31 (Clone 390, eBioscience). Podoplanin expression was revealed by hamster anti-mouse Podoplanin (Clone 8.1.1), followed by biotinylated anti-hamster-IgG antibody and streptavidin-PE (eBioscience). Stained cells were analyzed on a FACSCanto II using DIVA software (Becton Dickinson). Dead cells were excluded by a combination of light scatter and PI fluorescence. Cell duplets were excluded by forward scatter pulse width. Peripheral blood mononuclear cells were isolated by Ficoll–Histopaque (SIGMA) density-gradient centrifugation. Bone marrow cells were extracted from mouse femurs and tibiae by flushing. Tumor single cell suspensions were obtained by digestion for 45 minutes at 37°C using the following digestion buffers: TRAMP-C1: HEPES buffered saline, 0.1 mg/ml DNaseI (Roche), 1 mg/ml collagenase I (SIGMA); RT2: DMEM, 5% NU serum (Becton Dickinson), 0.2 mg/ml DNaseI, 1.2 U/ml DispaseII (Roche Applied Science).

### CD11b^+^ and CMP cell sorting and adoptive transfer

Bone marrow cells were extracted from femurs and tibiae of female C57BL/6-Tg(ACTB-EGFP) mice, washed in PBS/2% BSA, Fc blocked and stained with a phycoerythrin (PE)-conjugated monoclonal antibody against mouse CD11b (CALTAG) or, for CMP isolation, lineage markers, CD3, CD4 (Clone GK1.5), CD8 (Clone 53-6.7), Ter119 (Clone TER-119), B220 (Clone RA3-6B2), CD19, Gr-1 (Clone RB6-8C5), Sca-1 (Clone D7), IL7Rα (Clone A7R34) and Allophycocyanin-labeled anti-cKit (Clone 2B8) (all from eBioscience). CD11b^+^ GFP^+^ or CMP (lineage^−^/Sca-1^−^/IL7Rα^−^/cKit^+^) cells were sorted on a FACSAria (Becton Dickinson) with a purity >98%. 4×10^5^ CD11b^+^ or 4×10^4^ CMP were injected in the tail vein of semi-lethally (450 cGy) or non irradiated 9 week old RT2;VC mice, which were sacrificed 3 weeks after transplantation.

### Histological analysis

7 or 20 µm cryosections from pancreata or TRAMP-C1 tumors were prepared and stained as described [9]. Briefly, harvested tissues were fixed in 4% paraformaldehyde for 2 hours at 4°C, incubated in 30% sucrose overnight and then cryopreserved in OCT medium. Tissue sections were incubated at RT for 30 minutes with blocking buffer (5% goat serum in PBS) prior to overnight incubation at 4°C with the primary antibodies. When required, PBS/0.2% Triton-X-100 was used for permeabilization. The following primary antibodies were used at the dilutions specified in brackets: rat anti-mouse LYVE-1 (1∶200) (Clone ALY-7, MBL, Japan), rabbit anti-mouse LYVE-1 (1∶200) (Reliatech, Germany), rabbit anti-mouse Prox-1 (1∶100) (K. Alitalo, University of Helsinki), goat anti-human Prox-1 (1∶100) (R&D Systems), rabbit anti-Podoplanin (1∶100) (D. Kerjaschki, Medical University Vienna), hamster anti-mouse Podoplanin hybridoma supernatant (1∶20) (Clone 8.1.1), rat anti-mouse VE-Cadherin hybridoma supernatant (1∶50) (Clone B14, E. Dejana, University of Milano), rat anti-mouse F4/80 (1∶200) (Clone CI:A3-1, Serotec), rat anti-mouse CD11b (1∶100) (Clone M1/70.15, Serotec) and rat anti-mouse CD31 (1∶50) (Clone MEC 13.3, Pharmingen). Alexa Fluor 488-, 568- and 633-labeled secondary antibodies (Molecular Probes) were used (1∶400). Alexa Fluor 488-conjugated rabbit anti-GFP antibody (1∶500) (Molecular Probes) was employed for the detection of GFP. DAPI (SIGMA) was used for nuclear counterstaining. Sections were analyzed on a Nikon Diaphot 300 immunofluorescence microscope (Nikon) using Openlab 3.1.7. software (Improvision) or with a LSM 510 Meta confocal microscope using LSM software for 2D and 3D analysis (Zeiss). Videos were created using Imaris 6.1.1 software (Bitplane Scientific Solutions, Zurich, Switzerland).

### ClodroLip-mediated macrophage depletion

Eight week old RT2;VC mice were injected *i.p.* every 4 days for 4 weeks with 80 mg/kg body weight (first injection) or 40 mg/kg body weight (following injections) ClodroLip or with an equal volume of PBS as control. 2 days after the last injection, mice were sacrificed, pancreata were embedded in OCT and snap frozen in liquid nitrogen. Tumor macrophage depletion and tumor lymphatic vessel coverage were determined by immunofluorescence stainings with anti-F4/80 antibodies and anti-LYVE-1 antibodies, respectively, and ImageJ software (http://rsb.info.nih.gov/ij/). Statistical analysis and graphs were performed with GraphPad Prism software (GraphPad Software Inc.). Non-parametric Mann-Whitney tests were used to compare tumor lymphatic vessel coverage of treated versus control mice.

### Isolation of tumor-associated macrophages (TAM) and tumor cells

Single cell suspensions of tumors from 13–14 week old RT2;VC mice were obtained as described above, washed in FACS buffer (PBS/2% BSA/5 mM EDTA) and stained with anti-CD11b-PE and anti-CD31-APC. 20′000–50′000 CD11b^+^ cells (TAM) or CD11b^−^ CD31^−^ cells (tumor cells) were sorted on a FACSAria directly into TRIZOL reagent (Invitrogen).

### Quantitative RT–PCR

Total RNA was prepared using TRIZOL (in the case of RNA isolation from Matrigel cultures, two consecutive rounds of TRIZOL purification were performed), and reverse transcribed with random hexamer primers using M-MLV reverse transcriptase (SIGMA). cDNA was quantified on a ABI Prism 7000 light cycler (Applied Biosystems) using SYBR green PCR MasterMix (Fermentas) using the following primers: mVEGFC: fwd: 5′-AGCAGCCACAAACACCTTCTT-3′, rev: 5′-TCAAACAACGTCTTGCTGAGG-3′; mVEGFD: fwd: 5′-GCACCTCCTACATCTCCAAACAG-3′, rev: 5′-GGCAAGCACTTACAACCCGTAT-3′; mFGF1: fwd: 5′-CCGAAGGGCTTTTATACGG-3′, rev: 5′-TCTTGGAGGTGTAAGTGTTATAATGG-3′; mFGF2: fwd: 5′-CGGCTCTACTGCAAGAACG-3′, rev: 5′-TGCTTGGAGTTGTAGTTTGACG-3′; mFGFR1: fwd: 5′-TGTTTGACCGGATCTACACACA-3′, rev: 5′-CTCCCACAAGAGCACTCCAA-3′; mFGFR2: fwd: 5′-TCGCATTGGAGGCTATAAGG-3′, rev: 5′-CGGGACCACACTTTCCATAA-3′; mLYVE-1: fwd: 5′-GGTGTCCTGATTTGGAATGC-3′, rev: 5′- AGGAGTTAACCCAGGTGTCG -3′; mProx-1: fwd: 5′-AAGAGAGAGAGAAAGAGAGAGAGTGG-3′, rev: 5′-TGGGCACAGCTCAAGAATC-3′; mVEGF-R3: fwd: 5′-CGTGTGTGAAGTGCAGGATAGG-3′, rev: 5′-TCACTCACGTTCACCAGGAGGT-3′; mFoxC1: fwd: 5′-GCTTTCCTGCTCATTCGTCTT-3′, rev: 5′-AAATATCTTACAGGTGAGAGGCAAG-3′; mFoxC2: fwd: 5′-GACCCTAGCTCGCTGACG-3′, rev: 5′-CACCAGCCCTTCCGAGT-3′; mCD45: fwd: 5′-CAAAAGCAGATCGTCCGGA-3′, rev: 5′-TGTCGGCCGGGAGGTT-3′; mCX3CR1: fwd: 5′-AAGTTCCCTTCCCATCTGCT-3′, rev: 5′-CAAAATTCTCTAGATCCAGTTCAGG-3′; mRPL19: fwd: 5′-ATCCGCAAGCCTGTGACTGT-3′, rev: 5′-TCGGGCCAGGGTGTTTTT-3′. Ct values were normalized against ribosomal protein L19 (RPL19) and the levels of expression were presented as ΔΔCT or as mRNA copies per 1000 copies of RPL19 control mRNA.

### Tube formation assay using bone marrow-derived-macrophages

Bone marrow cells were extracted from femurs and tibiae of C57BL/6 or C57BL/6-Tg(ACTB-EGFP) mice and cultured on Teflon plates for 7 days in DMEM supplemented with 10% FBS, 2 mM glutamine, 100 units/ml penicillin and 30% L929 cell conditioned media containing M-CSF. Bone marrow-derived-macrophages were collected with PBS/1 mM EDTA. Matrigel (Becton Dickinson) was mixed 1∶1 with endothelial cell medium (EGM-2 MV, Cambrex) and allowed to solidify for 1 hour at 37°C in 8-chamber slides. 2−3×10^5^ bone marrow-derived macrophages or immortalized lymphatic endothelial cells (SV-LEC) or a mixture of 1.5×10^5^ cells each in EGM-2 MV supplemented with 1 µg/ml LPS were seeded onto the polymerized matrigel and tube formation was monitored up to 20 days. Immunofluorescence staining of cord-like structures was performed as described [50]. For time-lapse video microscopy, Hoechst labeled SV-LEC and GFP^+^ macrophages were co-cultured as described above and pictures were taken every 10 minutes for a period of 12 hours using a Zeiss Axiovert 35 M microscope (Zeiss), Princeton Instruments CCD camera and Metamorph Imaging software (Universal Imaging Corporation).

## Supporting Information

Movie S1 3D reconstitution of a 20 µm tissue section of a tumor, derived from a RT2;VC mouse transplanted with GFP labeled bone marrow. Arrow indicates a GFP+ cell (green) integrated in the Podoplanin+ (red) lymphatic vessel. Arrowhead indicates a GFP+ cell not completely integrated (noticeable only at certain angles). Ex: exocrine pancreas, L: lymphatic vessel lumen, T: tumor.
(2.86 MB MOV)

Movie S2 3D reconstitution of a 7 µm tissue section of a TRAMP-C1 tumor, grown in a GFP labeled bone marrow transplanted C57BL/6 mouse. Arrow indicates a GFP+ cell (green) integrated in the Podoplanin+ (red) lymphatic vessel.
(0.82 MB MOV)

Movie S3 Macrophages initiate lymphatic endothelial cell tube formation in in vitro co-culture. Time-lapse video microscopy after 24 h co-culture of SV-LEC and GFP+ macrophages on Matrigel. GFP+ macrophages were distinguished from SV-LEC by their bright signal in phase-contrast. The movie shows a GFP+ macrophage located at the rim of SV-LEC compact structure initiating outgrowth of a tube-like structure. As also visible in Figure S8, the SV-LEC follow the tube-leading macrophage in the newly forming tube-like structure.
(1.08 MB MOV)

Figure S1 Infiltration of transplanted BMDC in RT2 tumours. Lethally irradiated RT2 and RT2;VC mice were transplanted with GFP-labeled bone marrow, as indicated. Approximately 3–3.5% of tumour-constituting cells were GFP+ (green) and thus bone marrow-derived, and approximately 80% of GFP+ cells co-expressed the monocyte/macrophage marker F4/80 (red). White rectangles indicate area of higher magnification shown below, with merge picture on the left, F4/80 in the middle, and GFP on the right. F4/80+ macrophages are either donor-derived (co-expressing GFP, indicated by arrows) or host-derived (no GFP expression, arrowheads). 3 mice per genotype with 16–29 tumours each were analyzed. DAPI was used for nuclear counterstaining (blue). Scale bar: 50 µm.
(1.95 MB JPG)

Figure S2 BMDC do not integrate into lymphatic vessels surrounding normal islets. VC single-transgenic mice were transplanted with GFP-labeled bone marrow. Pancreatic sections of transplanted mice were stained for the lymphatic markers LYVE-1 (red) and GFP (green) and analyzed by confocal microscopy. Two representative pancreatic sections with islets of Langerhans are shown. No GFP+ cells were found integrated into islet-surrounding lymphatic structures. DAPI stains nuclei (blue). Scale bar: 20 µm.
(1.17 MB JPG)

Figure S3 FACS analysis of lineage tracing experiments. (A) FACS analysis of peripheral bood cells from a representative RT2;VC mouse reconstituted with bone marrow isolated from CX3CR1+/GFP mice indicates GFP expression mainly in CD11b+ cells. A minor fraction of CD19+ B-cells and CD3+ T-cells also expressed GFP. (B) FACS analysis of peripheral blood cells from CD11b-Cre;Z/EG mice transplanted with TRAMP-C1 tumours indicates effective Cre-mediated recombination and subsequent expression of GFP predominantly in F4/80+ monocytes and to much lower extent in B or T lymphocytes. (C) FACS analysis of peripheral blood cells from RT2;VC mice reconstituted with common myeloid progenitor (CMP) cells indicates that GFP+ cells are present within the CD11b+/F4/80+ monocyte fraction and the CD11b+/F4/80− granulocyte fraction but not in B or T lymphocytes.
(2.92 MB JPG)

Figure S4 Pancreatic sections of RT2;VC mice adoptively transferred with FACS-sorted GFP+ common myeloid progenitor cells (CMP) (3 mice) were stained for LYVE-1 or Podoplanin as well as for GFP and analyzed by confocal microscopy. Representative tumor sections are shown. Double positive cells for GFP (Green) and LYVE-1 or Podoplanin (red) are observed, demonstrating that CMP provide cells that incorporate into tumor lymphatics. DAPI stains nuclei (blue). Scale bars: 20 µm.
(1.54 MB JPG)

Figure S5 CD19+ B lymphocytes do not integrate into tumour-associated lymphatics. FACS sorted CD19+/GFP+ cells were adoptively transferred into semi-lethally irradiated RT2;VC mice (2 mice). 3 weeks after transfer, mice were sacrificed and tumour sections were stained for the lymphatic marker LYVE-1 (red) and for GFP (green) and analyzed by confocal microscopy. No GFP+ cells co-expressing LYVE-1 could be observed. DAPI was used for nuclear counterstaining (blue). Scale bars: 20 µm.
(1.37 MB JPG)

Figure S6 Tumors of non bone marrow-transplanted RT2;VC mice were enzymatically digested. Single cell suspension were stained for the pan-endothelial marker CD31, the lymphatic endothelial marker LYVE-1 and the myeloid marker CD11b and analyzed by FACS. 6.2+/−4.5% of CD31+/LYVE-1+ TLEC co-expressed CD11b.
(0.76 MB JPG)

Figure S7 Macrophage depletion does not affect tumor growth. RT2;VC mice were treated for 4 weeks either with PBS (vehicle control) or with ClodroLip in order to deplete intra- and peritumoral macrophages. Tumor volume has been determined as the total volume of tumors per mouse (A), tumor incidence is the number of tumors larger than 1 mm per mouse (B), and blood vessel density is the % area fraction of CD31 staining (C), as determined by ImageJ image analysis software. None of these parameters was significantly altered between ClodroLip and control-treated mice.
(1.16 MB JPG)

Figure S8 Macrophages initiate lymphatic endothelial cell tube formation in an in vitro co-culture system.
(0.97 MB JPG)

## References

[1] De Palma M, Venneri MA, Galli R, Sergi Sergi L, Politi LS (2005). Tie2 identifies a hematopoietic lineage of proangiogenic monocytes required for tumor vessel formation and a mesenchymal population of pericyte progenitors.. Cancer Cell.

[2] Grunewald M, Avraham I, Dor Y, Bachar-Lustig E, Itin A (2006). VEGF-induced adult neovascularization: recruitment, retention, and role of accessory cells.. Cell.

[3] Lyden D, Hattori K, Dias S, Costa C, Blaikie P (2001). Impaired recruitment of bone-marrow-derived endothelial and hematopoietic precursor cells blocks tumor angiogenesis and growth.. Nat Med.

[4] Coussens LM, Tinkle CL, Hanahan D, Werb Z (2000). MMP-9 supplied by bone marrow-derived cells contributes to skin carcinogenesis.. Cell.

[5] Cursiefen C, Chen L, Borges LP, Jackson D, Cao J (2004). VEGF-A stimulates lymphangiogenesis and hemangiogenesis in inflammatory neovascularization via macrophage recruitment.. J Clin Invest.

[6] Kaplan RN, Riba RD, Zacharoulis S, Bramley AH, Vincent L (2005). VEGFR1-positive haematopoietic bone marrow progenitors initiate the pre-metastatic niche.. Nature.

[7] Garcia-Barros M, Paris F, Cordon-Cardo C, Lyden D, Rafii S (2003). Tumor response to radiotherapy regulated by endothelial cell apoptosis.. Science.

[8] Purhonen S, Palm J, Rossi D, Kaskenpaa N, Rajantie I (2008). Bone marrow-derived circulating endothelial precursors do not contribute to vascular endothelium and are not needed for tumor growth.. Proc Natl Acad Sci U S A.

[9] Bailey AS, Willenbring H, Jiang S, Anderson DA, Schroeder DA (2006). Myeloid lineage progenitors give rise to vascular endothelium.. Proc Natl Acad Sci U S A.

[10] Jiang S, Bailey AS, Goldman DC, Swain JR, Wong MH (2008). Hematopoietic stem cells contribute to lymphatic endothelium.. PLoS ONE.

[11] Ichise H, Ichise T, Ohtani O, Yoshida N (2009). Phospholipase Cgamma2 is necessary for separation of blood and lymphatic vasculature in mice.. Development.

[12] Sebzda E, Hibbard C, Sweeney S, Abtahian F, Bezman N (2006). Syk and Slp-76 mutant mice reveal a cell-autonomous hematopoietic cell contribution to vascular development.. Dev Cell.

[13] Religa P, Cao R, Bjorndahl M, Zhou Z, Zhu Z (2005). Presence of bone marrow-derived circulating progenitor endothelial cells in the newly formed lymphatic vessels.. Blood.

[14] Kerjaschki D, Huttary N, Raab I, Regele H, Bojarski-Nagy K (2006). Lymphatic endothelial progenitor cells contribute to de novo lymphangiogenesis in human renal transplants.. Nat Med.

[15] Hamrah P, Chen L, Zhang Q, Dana MR (2003). Novel expression of vascular endothelial growth factor receptor (VEGFR)-3 and VEGF-C on corneal dendritic cells.. Am J Pathol.

[16] Maruyama K, Ii M, Cursiefen C, Jackson DG, Keino H (2005). Inflammation-induced lymphangiogenesis in the cornea arises from CD11b-positive macrophages.. J Clin Invest.

[17] Maruyama K, Asai J, Ii M, Thorne T, Losordo DW (2007). Decreased macrophage number and activation lead to reduced lymphatic vessel formation and contribute to impaired diabetic wound healing.. Am J Pathol.

[18] He Y, Rajantie I, Ilmonen M, Makinen T, Karkkainen MJ (2004). Preexisting lymphatic endothelium but not endothelial progenitor cells are essential for tumor lymphangiogenesis and lymphatic metastasis.. Cancer Res.

[19] Hanahan D (1985). Heritable formation of pancreatic beta-cell tumours in transgenic mice expressing recombinant insulin/simian virus 40 oncogenes.. Nature.

[20] Bergers G, Brekken R, McMahon G, Vu TH, Itoh T (2000). Matrix metalloproteinase-9 triggers the angiogenic switch during carcinogenesis.. Nat Cell Biol.

[21] Nozawa H, Chiu C, Hanahan D (2006). Infiltrating neutrophils mediate the initial angiogenic switch in a mouse model of multistage carcinogenesis.. Proc Natl Acad Sci U S A.

[22] Perl AK, Wilgenbus P, Dahl U, Semb H, Christofori G (1998). A causal role for E-cadherin in the transition from adenoma to carcinoma.. Nature.

[23] Mandriota SJ, Jussila L, Jeltsch M, Compagni A, Baetens D (2001). Vascular endothelial growth factor-C-mediated lymphangiogenesis promotes tumour metastasis.. Embo J.

[24] Cho CH, Koh YJ, Han J, Sung HK, Jong Lee H (2007). Angiogenic role of LYVE-1-positive macrophages in adipose tissue.. Circ Res.

[25] Schledzewski K, Falkowski M, Moldenhauer G, Metharom P, Kzhyshkowska J (2006). Lymphatic endothelium-specific hyaluronan receptor LYVE-1 is expressed by stabilin-1+, F4/80+, CD11b+ macrophages in malignant tumours and wound healing tissue in vivo and in bone marrow cultures in vitro: implications for the assessment of lymphangiogenesis.. J Pathol.

[26] Baluk P, Fuxe J, Hashizume H, Romano T, Lashnits E (2007). Functionally specialized junctions between endothelial cells of lymphatic vessels.. J Exp Med.

[27] Gingrich JR, Barrios RJ, Morton RA, Boyce BF, DeMayo FJ (1996). Metastatic prostate cancer in a transgenic mouse.. Cancer Res.

[28] Ozerdem U (2006). Targeting of pericytes diminishes neovascularization and lymphangiogenesis in prostate cancer.. Prostate.

[29] Jung S, Aliberti J, Graemmel P, Sunshine MJ, Kreutzberg GW (2000). Analysis of fractalkine receptor CX(3)CR1 function by targeted deletion and green fluorescent protein reporter gene insertion.. Mol Cell Biol.

[30] Novak A, Guo C, Yang W, Nagy A, Lobe CG (2000). Z/EG, a double reporter mouse line that expresses enhanced green fluorescent protein upon Cre-mediated excision.. Genesis.

[31] Ferron M, Vacher J (2005). Targeted expression of Cre recombinase in macrophages and osteoclasts in transgenic mice.. Genesis.

[32] McDonnell CO, Bouchier-Hayes DJ, Toomey D, Foley D, Kay EW (2003). Effect of neoadjuvant chemoradiotherapy on angiogenesis in oesophageal cancer.. Br J Surg.

[33] Akashi K, Traver D, Miyamoto T, Weissman IL (2000). A clonogenic common myeloid progenitor that gives rise to all myeloid lineages.. Nature.

[34] Van Rooijen N (1989). The liposome-mediated macrophage ‘suicide’ technique.. J Immunol Methods.

[35] Van Rooijen N, Sanders A (1994). Liposome mediated depletion of macrophages: mechanism of action, preparation of liposomes and applications.. J Immunol Methods.

[36] Zeisberger SM, Odermatt B, Marty C, Zehnder-Fjallman AH, Ballmer-Hofer K (2006). Clodronate-liposome-mediated depletion of tumour-associated macrophages: a new and highly effective antiangiogenic therapy approach.. Br J Cancer.

[37] Gatfield J, Pieters J (2000). Essential role for cholesterol in entry of mycobacteria into macrophages.. Science.

[38] Ando T, Jordan P, Joh T, Wang Y, Jennings MH (2005). Isolation and characterization of a novel mouse lymphatic endothelial cell line: SV-LEC.. Lymphat Res Biol.

[39] Bertolini F, Shaked Y, Mancuso P, Kerbel RS (2006). The multifaceted circulating endothelial cell in cancer: towards marker and target identification.. Nat Rev Cancer.

[40] Gregory CA, Ylostalo J, Prockop DJ (2005). Adult bone marrow stem/progenitor cells (MSCs) are preconditioned by microenvironmental “niches” in culture: a two-stage hypothesis for regulation of MSC fate.. Sci STKE.

[41] Eilken HM, Nishikawa S, Schroeder T (2009). Continuous single-cell imaging of blood generation from haemogenic endothelium.. Nature.

[42] Lancrin C, Sroczynska P, Stephenson C, Allen T, Kouskoff V (2009). The haemangioblast generates haematopoietic cells through a haemogenic endothelium stage.. Nature.

[43] Chen MJ, Yokomizo T, Zeigler BM, Dzierzak E, Speck NA (2009). Runx1 is required for the endothelial to haematopoietic cell transition but not thereafter.. Nature.

[44] Loomans CJ, Wan H, de Crom R, van Haperen R, de Boer HC (2006). Angiogenic murine endothelial progenitor cells are derived from a myeloid bone marrow fraction and can be identified by endothelial NO synthase expression.. Arterioscler Thromb Vasc Biol.

[45] Abtahian F, Guerriero A, Sebzda E, Lu MM, Zhou R (2003). Regulation of blood and lymphatic vascular separation by signaling proteins SLP-76 and Syk.. Science.

[46] Gannon G, Mandriota SJ, Cui L, Baetens D, Pepper MS (2002). Overexpression of vascular endothelial growth factor-A165 enhances tumor angiogenesis but not metastasis during beta-cell carcinogenesis.. Cancer Res.

[47] Okabe M, Ikawa M, Kominami K, Nakanishi T, Nishimune Y (1997). ‘Green mice’ as a source of ubiquitous green cells.. FEBS Lett.

[48] Benard A, Ceredig R, Rolink AG (2006). Regulatory T cells control autoimmunity following syngeneic bone marrow transplantation.. Eur J Immunol.

[49] Foster BA, Gingrich JR, Kwon ED, Madias C, Greenberg NM (1997). Characterization of prostatic epithelial cell lines derived from transgenic adenocarcinoma of the mouse prostate (TRAMP) model.. Cancer Res.

[50] Debnath J, Muthuswamy SK, Brugge JS (2003). Morphogenesis and oncogenesis of MCF-10A mammary epithelial acini grown in three-dimensional basement membrane cultures.. Methods.

