# Supplementary figures and images for: Myeloid Cells Contribute to Tumor Lymphangiogenesis
Source: PLoS One. 2009 Sep 17;4(9):e7067. doi: 10.1371/journal.pone.0007067 (PMC2738969; doi:10.1371/journal.pone.0007067)

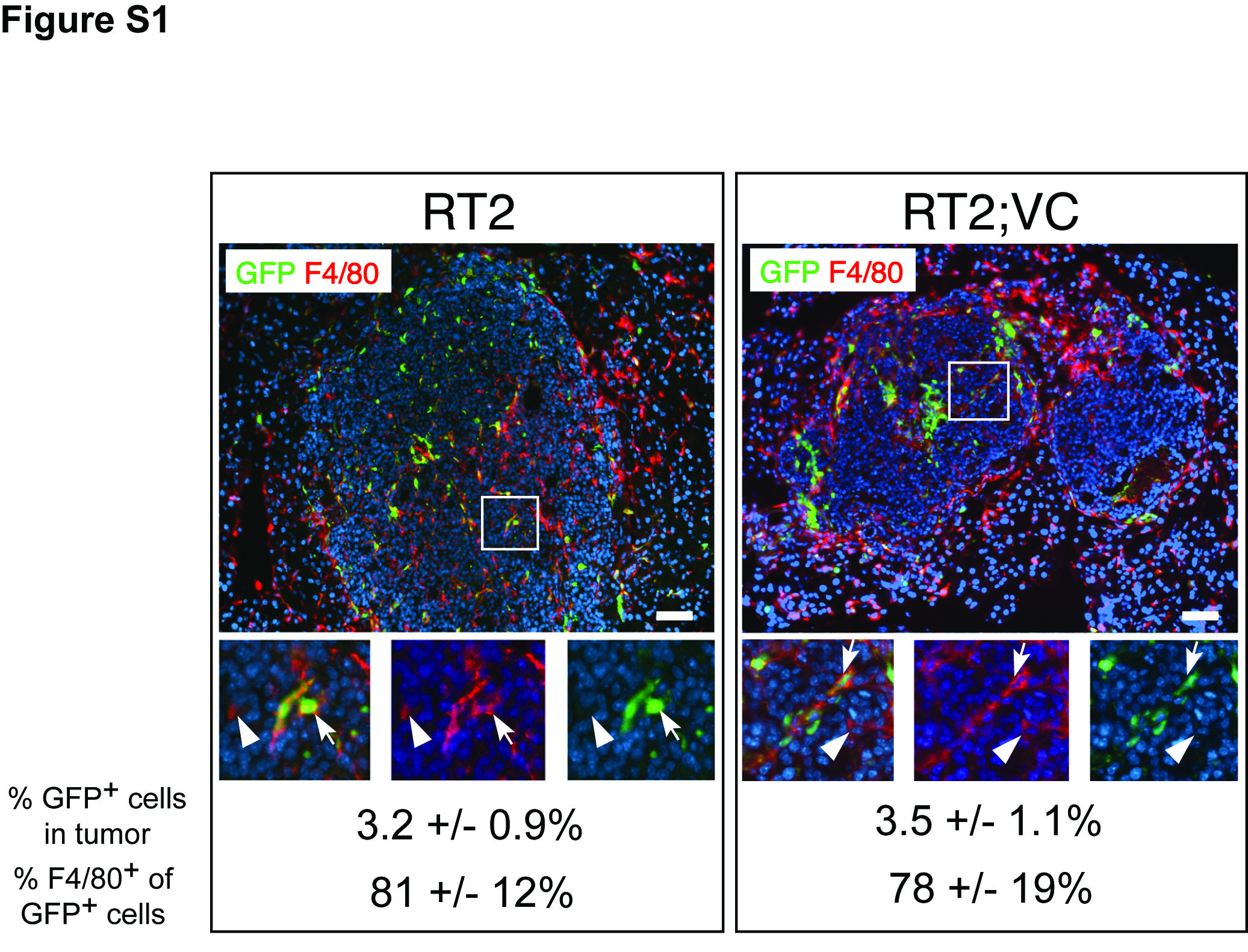

Supplement: Figure S1 — Infiltration of transplanted BMDC in RT2 tumours. Lethally irradiated RT2 and RT2;VC mice were transplanted with GFP-labeled bone marrow, as indicated. Approximately 3–3.5% of tumour-constituting cells were GFP+ (green) and thus bone marrow-derived, and approximately 80% of GFP+ cells co-expressed the monocyte/macrophage marker F4/80 (red). White rectangles indicate area of higher magnification shown below, with merge picture on the left, F4/80 in the middle, and GFP on the right. F4/80+ macrophages are either donor-derived (co-expressing GFP, indicated by arrows) or host-derived (no GFP expression, arrowheads). 3 mice per genotype with 16–29 tumours each were analyzed. DAPI was used for nuclear counterstaining (blue). Scale bar: 50 µm. (1.95 MB JPG) [file pone.0007067.s004.jpg]

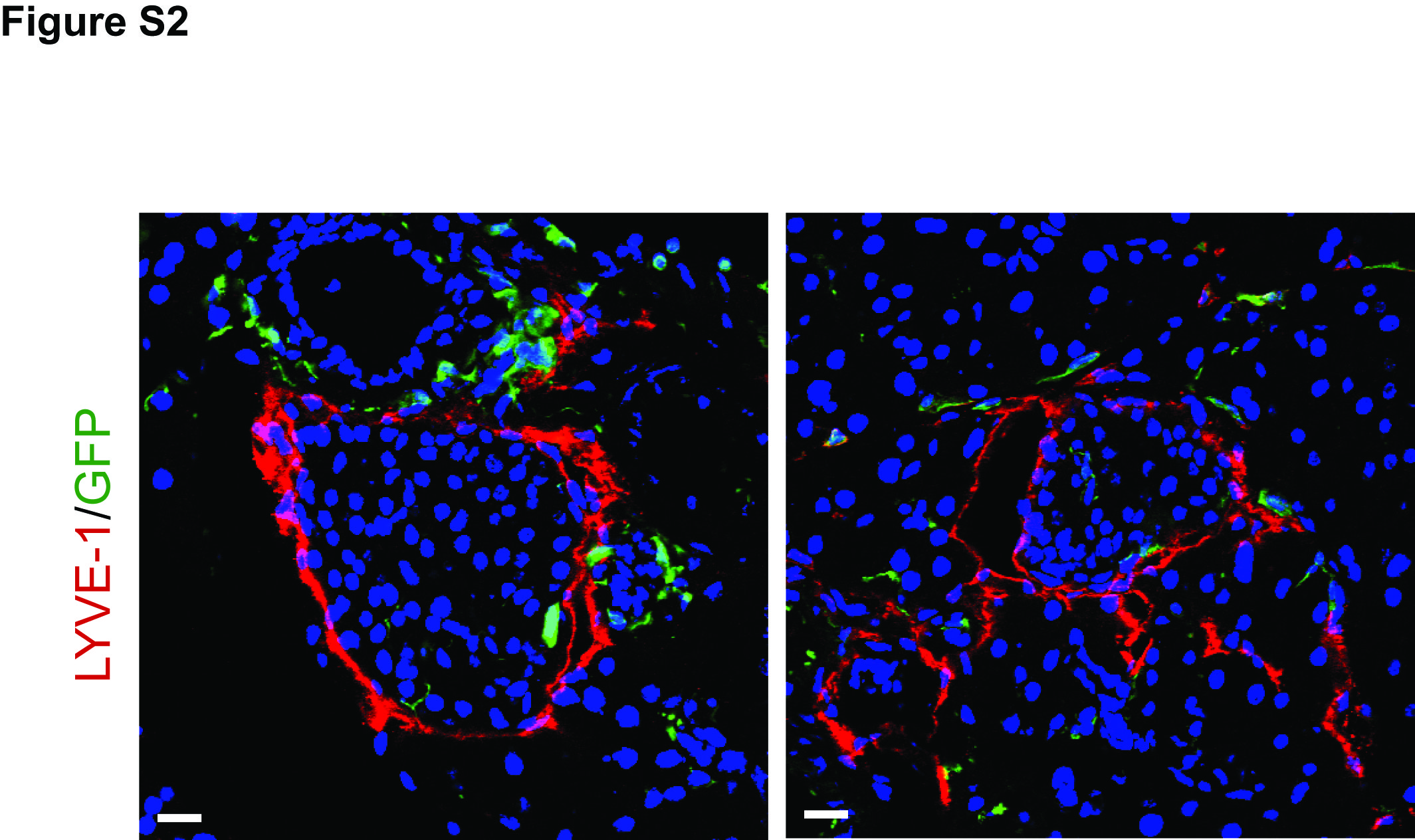

Supplement: Figure S2 — BMDC do not integrate into lymphatic vessels surrounding normal islets. VC single-transgenic mice were transplanted with GFP-labeled bone marrow. Pancreatic sections of transplanted mice were stained for the lymphatic markers LYVE-1 (red) and GFP (green) and analyzed by confocal microscopy. Two representative pancreatic sections with islets of Langerhans are shown. No GFP+ cells were found integrated into islet-surrounding lymphatic structures. DAPI stains nuclei (blue). Scale bar: 20 µm. (1.17 MB JPG) [file pone.0007067.s005.jpg]

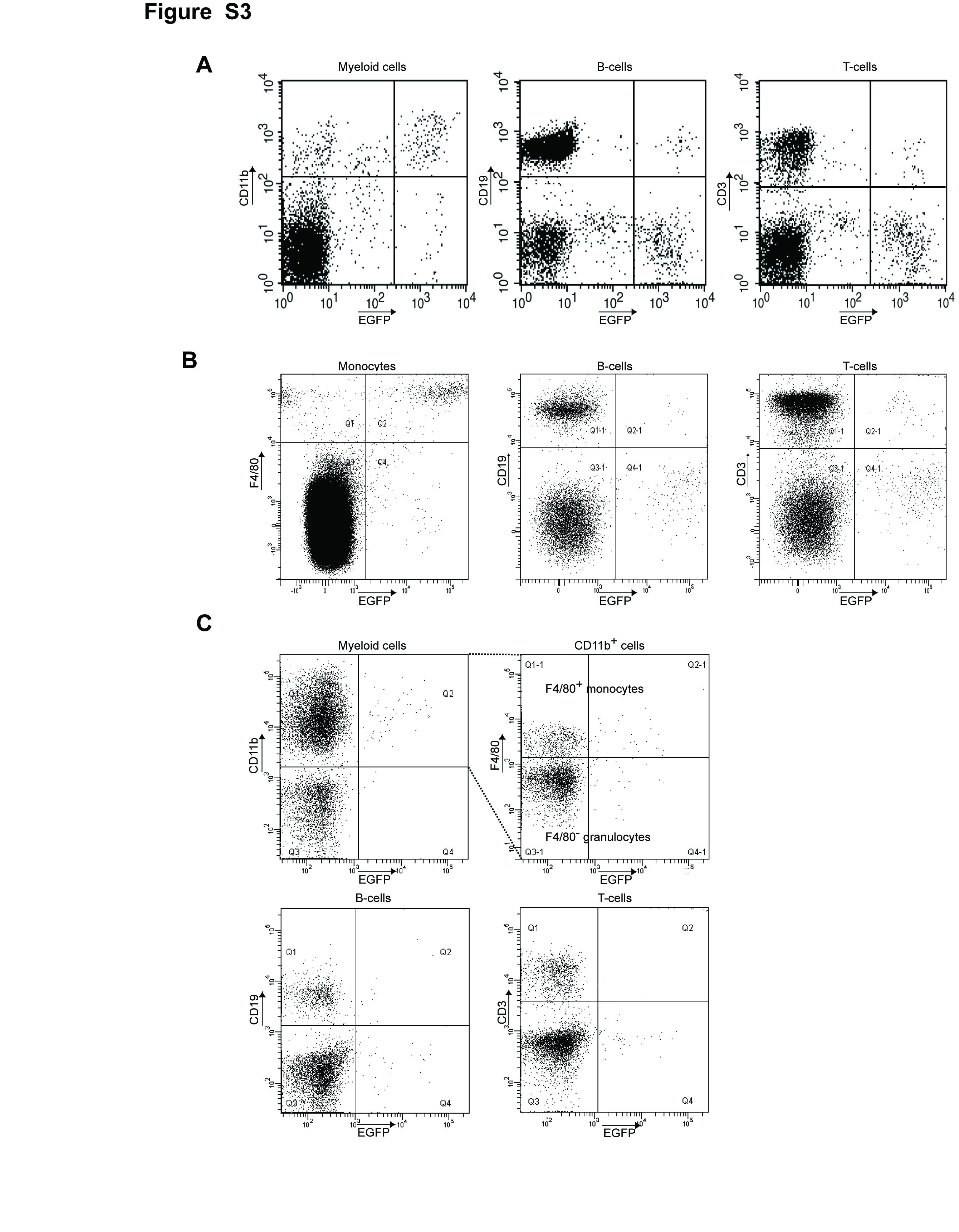

Supplement: Figure S3 — FACS analysis of lineage tracing experiments. (A) FACS analysis of peripheral bood cells from a representative RT2;VC mouse reconstituted with bone marrow isolated from CX3CR1+/GFP mice indicates GFP expression mainly in CD11b+ cells. A minor fraction of CD19+ B-cells and CD3+ T-cells also expressed GFP. (B) FACS analysis of peripheral blood cells from CD11b-Cre;Z/EG mice transplanted with TRAMP-C1 tumours indicates effective Cre-mediated recombination and subsequent expression of GFP predominantly in F4/80+ monocytes and to much lower extent in B or T lymphocytes. (C) FACS analysis of peripheral blood cells from RT2;VC mice reconstituted with common myeloid progenitor (CMP) cells indicates that GFP+ cells are present within the CD11b+/F4/80+ monocyte fraction and the CD11b+/F4/80− granulocyte fraction but not in B or T lymphocytes. (2.92 MB JPG) [file pone.0007067.s006.jpg]

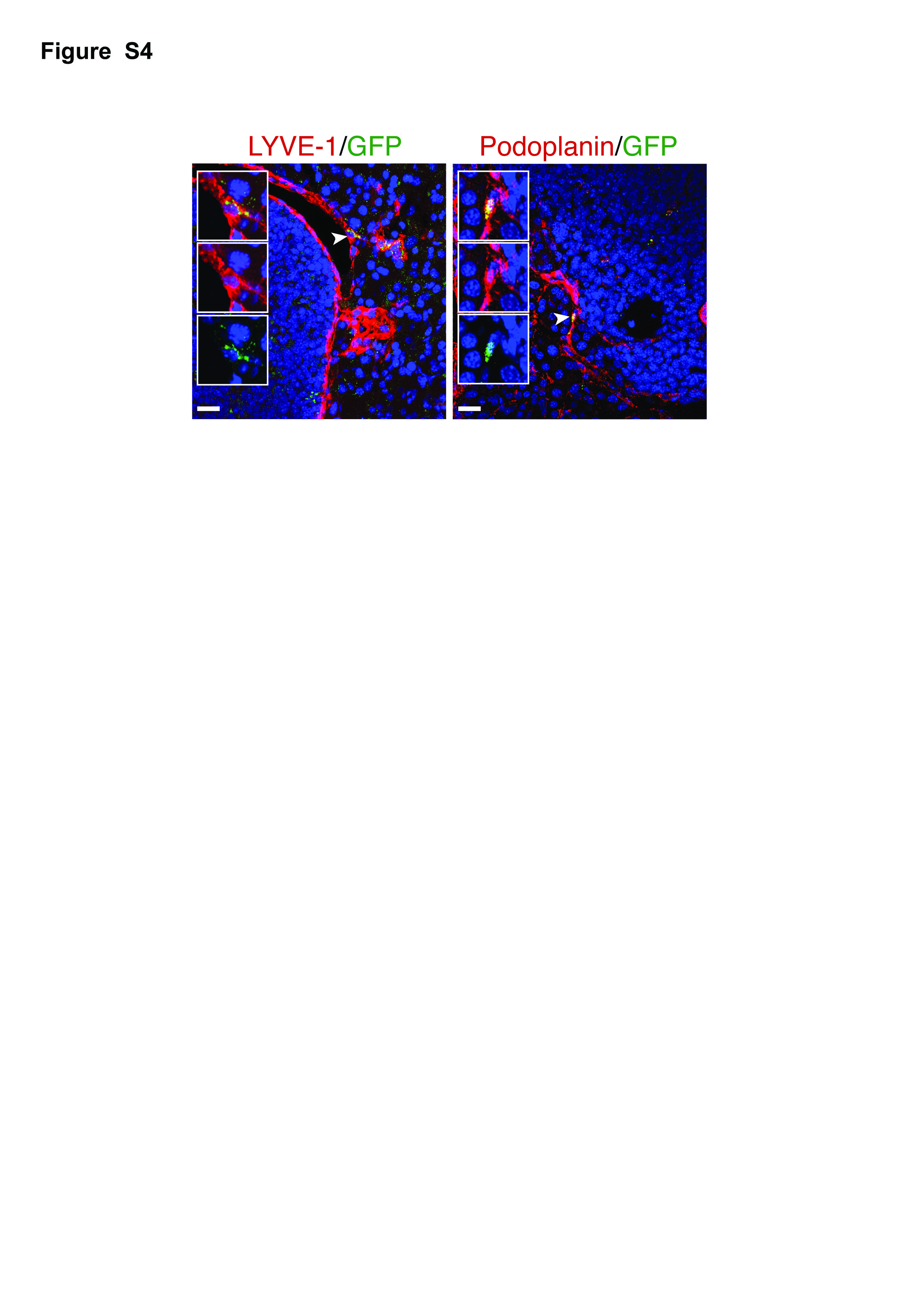

Supplement: Figure S4 — Pancreatic sections of RT2;VC mice adoptively transferred with FACS-sorted GFP+ common myeloid progenitor cells (CMP) (3 mice) were stained for LYVE-1 or Podoplanin as well as for GFP and analyzed by confocal microscopy. Representative tumor sections are shown. Double positive cells for GFP (Green) and LYVE-1 or Podoplanin (red) are observed, demonstrating that CMP provide cells that incorporate into tumor lymphatics. DAPI stains nuclei (blue). Scale bars: 20 µm. (1.54 MB JPG) [file pone.0007067.s007.jpg]

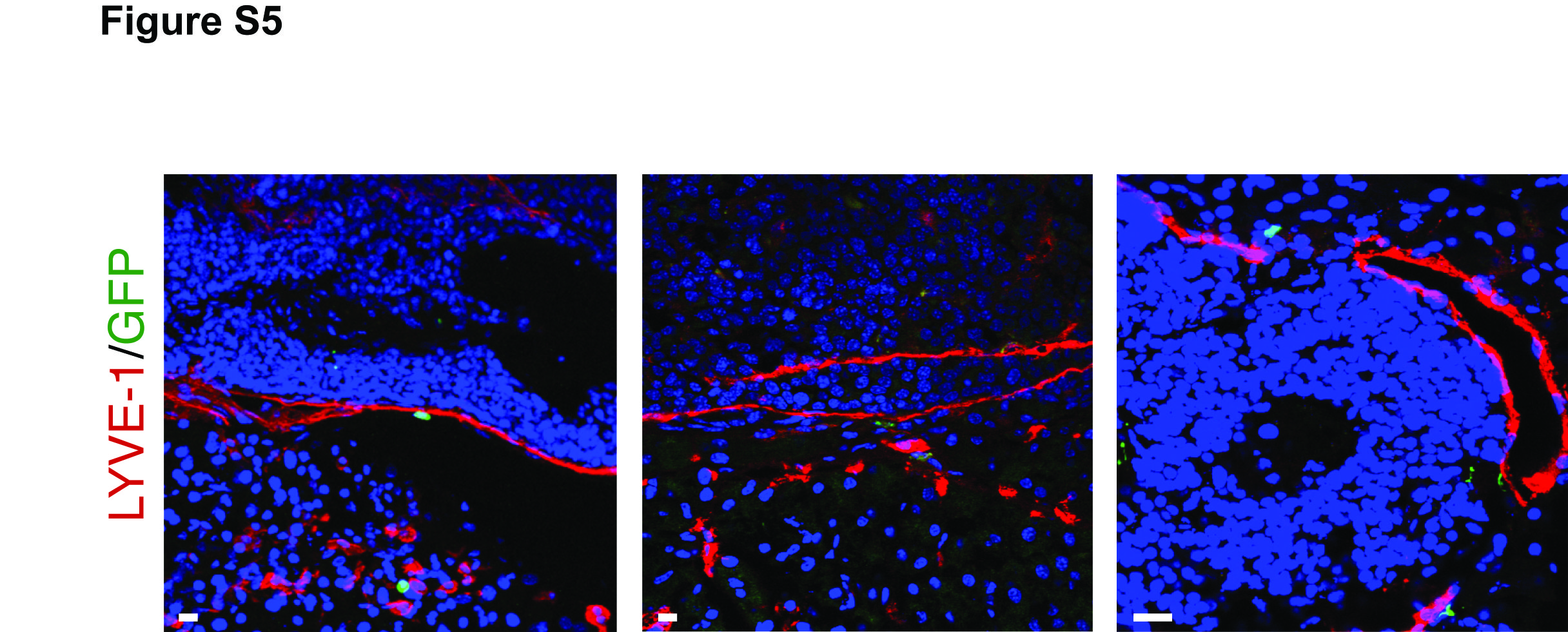

Supplement: Figure S5 — CD19+ B lymphocytes do not integrate into tumour-associated lymphatics. FACS sorted CD19+/GFP+ cells were adoptively transferred into semi-lethally irradiated RT2;VC mice (2 mice). 3 weeks after transfer, mice were sacrificed and tumour sections were stained for the lymphatic marker LYVE-1 (red) and for GFP (green) and analyzed by confocal microscopy. No GFP+ cells co-expressing LYVE-1 could be observed. DAPI was used for nuclear counterstaining (blue). Scale bars: 20 µm. (1.37 MB JPG) [file pone.0007067.s008.jpg]

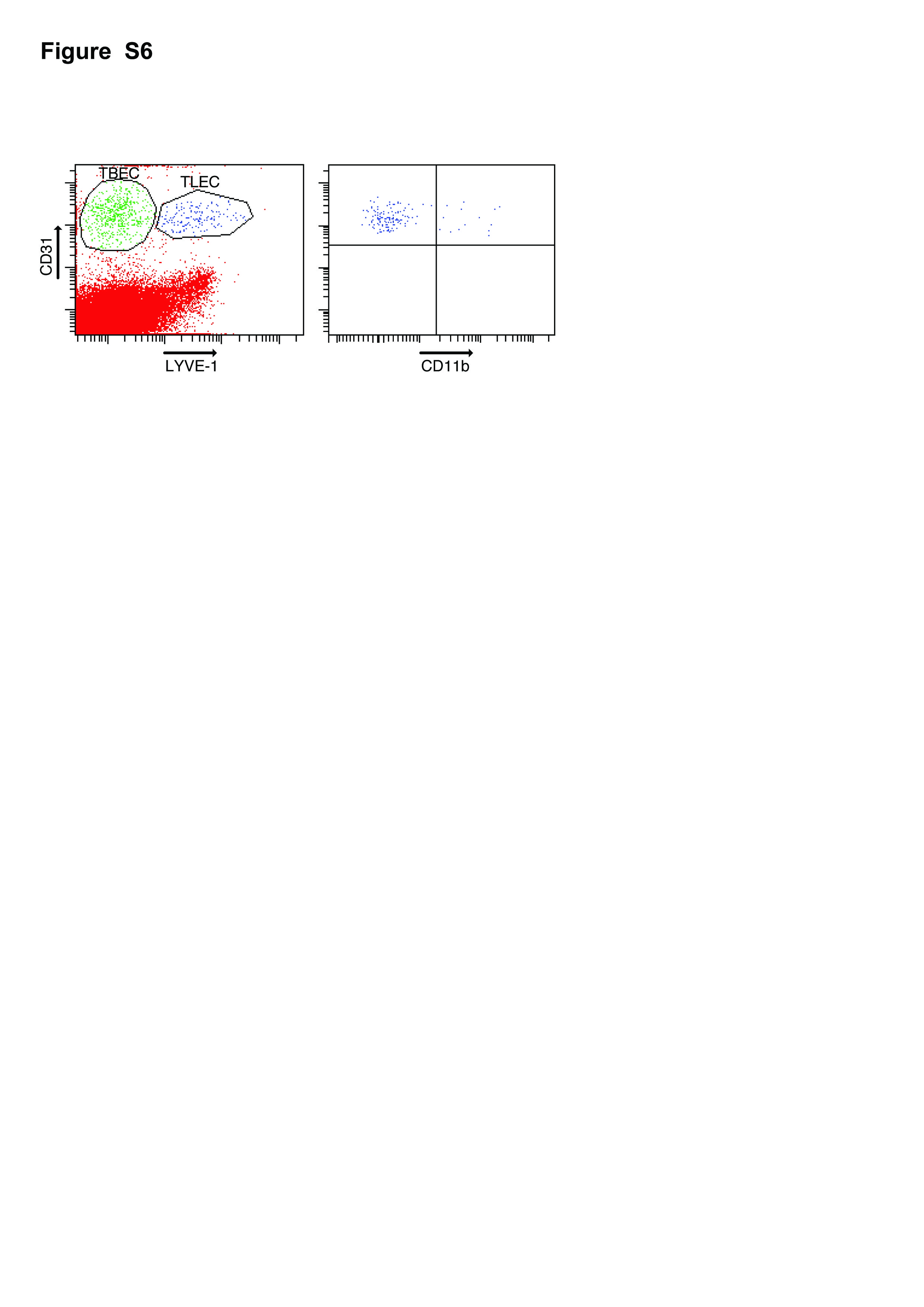

Supplement: Figure S6 — Tumors of non bone marrow-transplanted RT2;VC mice were enzymatically digested. Single cell suspension were stained for the pan-endothelial marker CD31, the lymphatic endothelial marker LYVE-1 and the myeloid marker CD11b and analyzed by FACS. 6.2+/−4.5% of CD31+/LYVE-1+ TLEC co-expressed CD11b. (0.76 MB JPG) [file pone.0007067.s009.jpg]

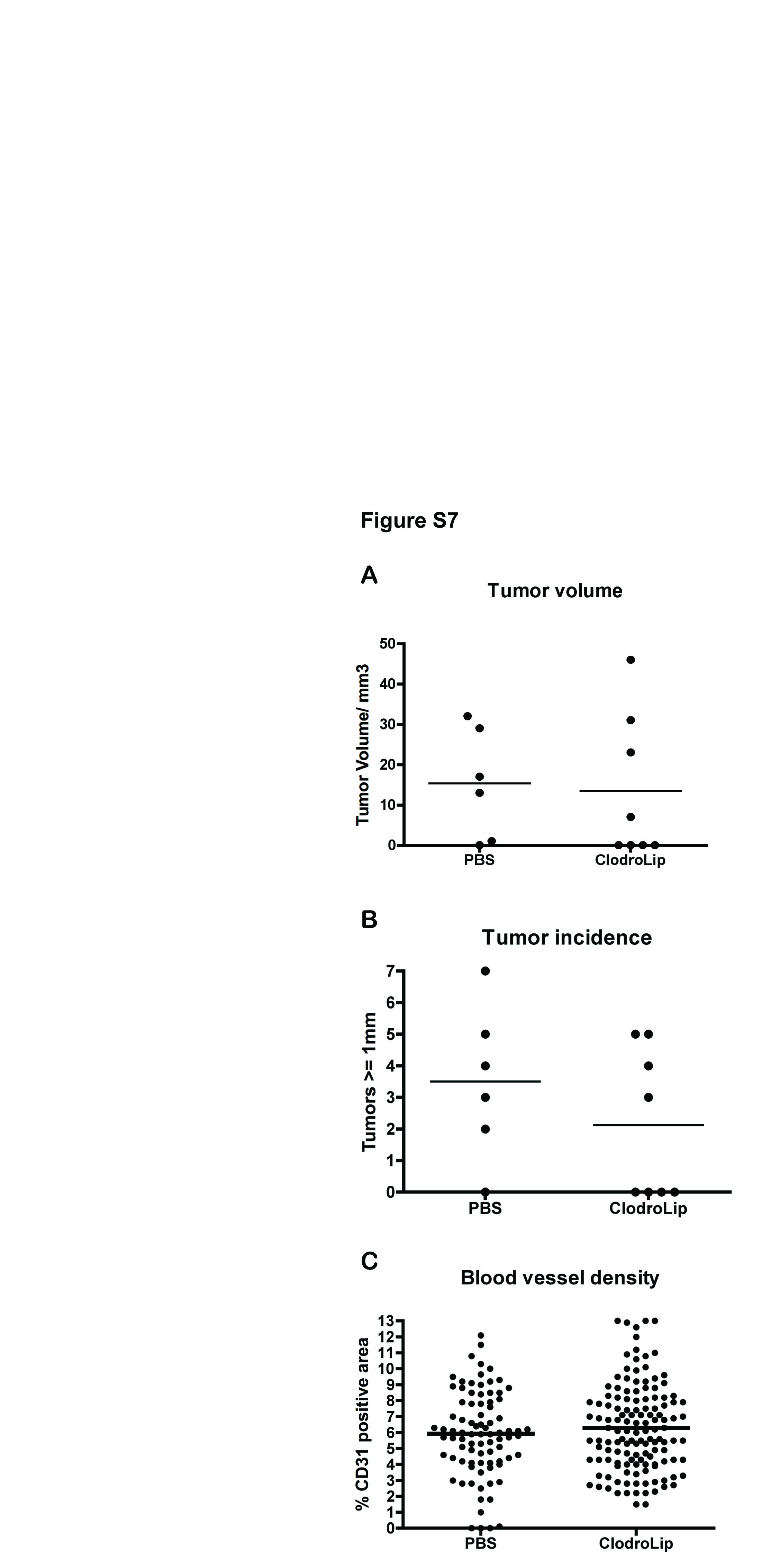

Supplement: Figure S7 — Macrophage depletion does not affect tumor growth. RT2;VC mice were treated for 4 weeks either with PBS (vehicle control) or with ClodroLip in order to deplete intra- and peritumoral macrophages. Tumor volume has been determined as the total volume of tumors per mouse (A), tumor incidence is the number of tumors larger than 1 mm per mouse (B), and blood vessel density is the % area fraction of CD31 staining (C), as determined by ImageJ image analysis software. None of these parameters was significantly altered between ClodroLip and control-treated mice. (1.16 MB JPG) [file pone.0007067.s010.jpg]

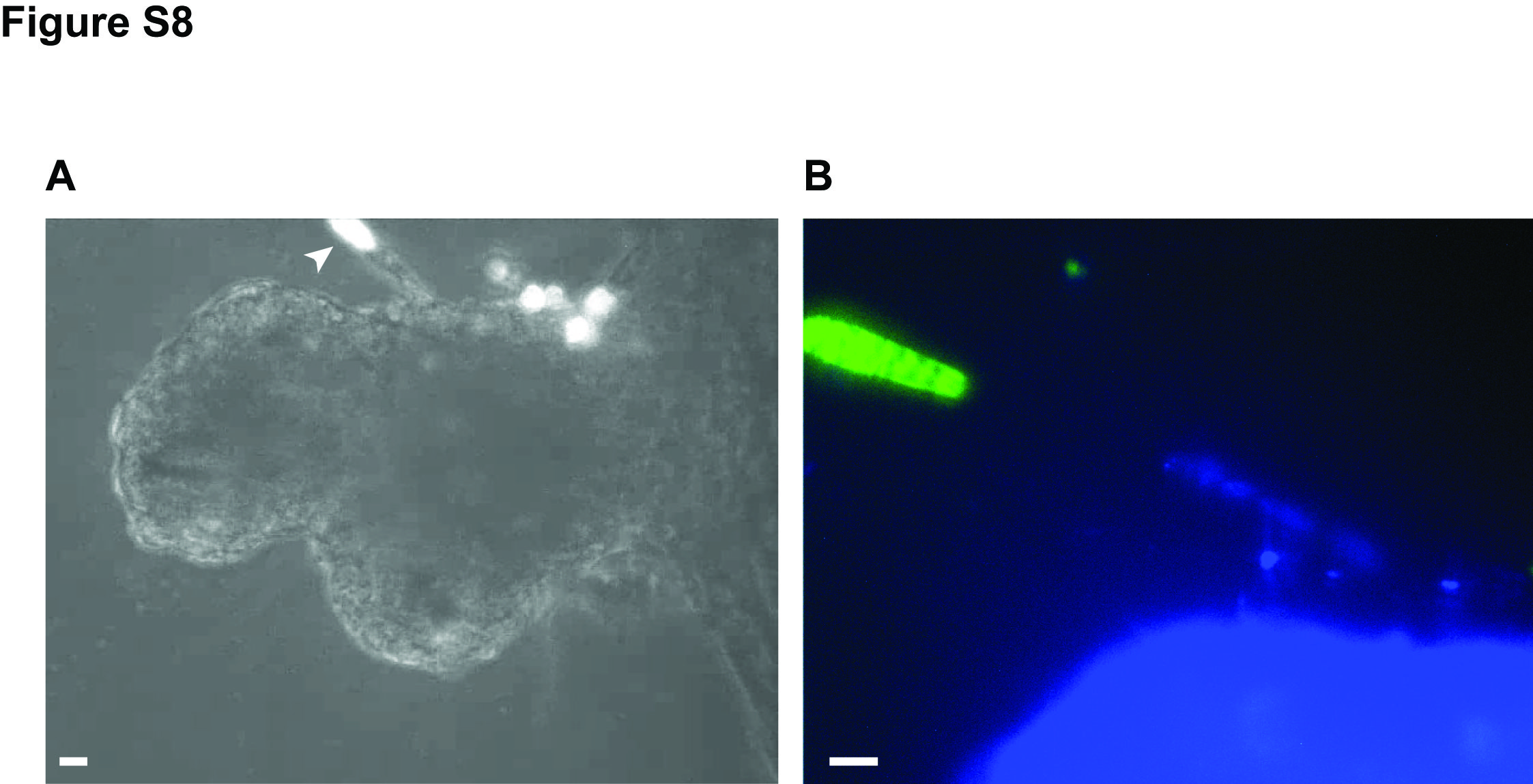

Supplement: Figure S8 — Macrophages initiate lymphatic endothelial cell tube formation in an in vitro co-culture system. (0.97 MB JPG) [file pone.0007067.s011.jpg]
